# MEG–EEG Information Fusion and Electromagnetic Source Imaging: From Theory to Clinical Application in Epilepsy

**DOI:** 10.1007/s10548-015-0437-3

**Published:** 2015-05-28

**Authors:** Rasheda Arman Chowdhury, Younes Zerouali, Tanguy Hedrich, Marcel Heers, Eliane Kobayashi, Jean-Marc Lina, Christophe Grova

**Affiliations:** Multimodal Functional Imaging Lab, Biomedical Engineering Department, McGill University, Duff Medical Building, 3775, rue University, Room 316, Montreal, QC H3A 2B4 Canada; Ecole de Technologie Supérieure, Montreal, Canada; Neurology and Neurosurgery Department, Montreal Neurological Institute, McGill University, Montreal, Canada; Centre de Recherches Mathématiques, Université de Montréal, Montreal, Canada; Physics Department and PERFORM Centre, Concordia University, Montreal, Canada; Epilepsy Center, University Hospital Freiburg, Freiburg, Germany

**Keywords:** Fusion, Electro-encephalography, Magneto-encephalography, Inter-ictal epileptic discharges, Spatio-temporal propagation, Maximum entropy on the mean framework

## Abstract

The purpose of this study is to develop and quantitatively assess whether fusion of EEG and MEG (MEEG) data within the maximum entropy on the mean (MEM) framework increases the spatial accuracy of source localization, by yielding better recovery of the spatial extent and propagation pathway of the underlying generators of inter-ictal epileptic discharges (IEDs). The key element in this study is the integration of the complementary information from EEG and MEG data within the MEM framework. MEEG was compared with EEG and MEG when localizing single transient IEDs. The fusion approach was evaluated using realistic simulation models involving one or two spatially extended sources mimicking propagation patterns of IEDs. We also assessed the impact of the number of EEG electrodes required for an efficient EEG–MEG fusion. MEM was compared with minimum norm estimate, dynamic statistical parametric mapping, and standardized low-resolution electromagnetic tomography. The fusion approach was finally assessed on real epileptic data recorded from two patients showing IEDs simultaneously in EEG and MEG. Overall the localization of MEEG data using MEM provided better recovery of the source spatial extent, more sensitivity to the source depth and more accurate detection of the onset and propagation of IEDs than EEG or MEG alone. MEM was more accurate than the other methods. MEEG proved more robust than EEG and MEG for single IED localization in low signal-to-noise ratio conditions. We also showed that only few EEG electrodes are required to bring additional relevant information to MEG during MEM fusion.

**Electronic supplementary material:**

The online version of this article (doi:10.1007/s10548-015-0437-3) contains supplementary material, which is available to authorized users.

## Introduction

A successful pre-surgical evaluation in epilepsy entails the accurate detection of the onset of epileptic discharges, their spatial extent and propagation patterns (Stefan 2009; Tanaka and Stufflebeam 2014). Inter-ictal epileptic discharges (IEDs), occurring between seizures in epilepsy, are commonly used as markers of epilepsy (Staley and Dudek 2006). These are spontaneous transient activities that are clearly distinguishable from background activity. The high temporal resolution of electro-encephalography (EEG) and magneto-encephalography (MEG) allows the detection of the fast propagating IEDs more efficiently than other imaging techniques (Stefan 2009; Ebersole and Ebersole 2010). MEG can detect epileptic activity from background activities when a cortical area greater than 4 cm^2^ is synchronously involved (Mikuni et al. 1997). EEG requires the activation of a larger region of the cortex (at least 10 cm^2^) to detect epileptic activity on the scalp recordings (Ebersole 1997; Tao et al. 2007; Von Ellenrieder et al. 2014). Source analysis of EEG and MEG data is commonly used to localize the generators of brain activities that are detectable on the scalp (Stefan et al. 2003; Knowlton and Shih 2004; Noachtar and Rémi 2009; Wendel et al. 2009). Spatio-temporal source analysis of EEG and MEG data may be useful for accurate detection and estimation of propagation patterns of epileptic discharges (Tanaka et al. 2010, 2014). In order to detect the onset and propagation patterns of IEDs, source localization of single spike is more appropriate than averaged spike. Indeed, averaging spikes may enhance the signal-to-noise ratio but the differences in the origin between single spikes may get lost in the averaging process (Bast et al. 2004, 2006). EEG and MEG are sensitive to different aspects of neuronal activity (Cohen and Cuffin, 1983; Sutherling et al. 1987; Hämäläinen et al. 1993; Baumgartner and Pataraia 2006; Funke et al. 2009; Yu et al. 2010; Haueisen et al. 2012). Integrating these two modalities can bring in complementary information thereby allowing better accuracy in source imaging. Symmetrical fusion of EEG and MEG data is possible since the two modalities can relate to the same neuronal dynamics (temporal information) when acquired simultaneously (Molins et al. 2008).

Several studies have reported the added value of combining the complementarities of EEG and MEG data when performing source localization. These so-called EEG–MEG fusion methods allows improving the spatial resolution of source analysis by increasing the number of recording channels (EEG electrodes + MEG sensors) and the overall head surface coverage. Using single equivalent current dipole (ECD) approach on simulated EEG/MEG and electrical median nerve stimulation data, Fuchs et al. 1998 suggested that deep sources mainly contribute to EEG data while superficial and tangential sources contribute mainly to MEG data. Baillet et al. (1999) proposed a joint EEG/MEG analysis, aiming at minimizing the mutual information between the two modalities, thus enhancing their respective complementarities. This EEG/MEG fusion strategy demonstrated reduced sensitivity to noise and improved localization accuracy. Using L2-based minimum norm estimate (MNE) and its variants, such as dynamic Statistical Parametric Mapping (dSPM), several studies demonstrated the added value of fusing EEG/MEG data using either simulated data (Liu et al. 2002), visual evoked responses (Sharon et al. 2007) and electrical median nerve stimulation (Molins et al. 2008). The advantage of combining EEG and MEG data was also evaluated using other inverse operators, such as sparse source reconstruction (Ding and Yuan 2013) on simulated data, linearly constrained minimum variance beamformer approach on simulated and auditory data (Hong et al. 2013) or Multiple Sparse Prior methods on face evoked responses (Henson et al. 2009). However, to the best of our knowledge, there exists no prior study that performed source analysis using EEG/MEG fusion data to optimize the source localization of spatially extended generators of propagating epileptic discharges.

ECD solutions have been extensively used for localizing the sources of focal interictal spikes but distributed source localization methods are ideal for estimating distributed network of brain activity seen during most IEDs (Barkley and Baumgartner 2003; Kobayashi et al. 2005). Some of the well-known and widely used distributed methods are MNE (Hämäläinen and Ilmoniemi 1994) and low resolution electromagnetic tomography (LORETA) (Pascual-Marqui et al. 1994). We proposed the maximum entropy on the mean (MEM) (Amblard et al. 2004) as an interesting framework with good sensitivity in recovering the spatial extent of the sources, when using simulated EEG data (Grova et al. 2006), simulated MEG data (Lina et al. 2012; Chowdhury et al. 2013), when comparing EEG/MEG sources to fMRI BOLD responses to epileptic discharges (Grova et al. 2008; Heers et al. 2014) and when comparing EEG/MEG sources to intracranial EEG findings (Heers et al. 2015). When applied to EEG or MEG data, MEM proved to be more accurate in recovering the source spatial extent, than MNE, LORETA and their variants within the hierarchical Bayesian framework (Friston et al. 2008). Therefore, the purpose of this study is to assess whether symmetrical fusion of EEG and MEG data within the MEM framework increases the spatial accuracy of the localization, by yielding better recovery of the spatial extent and propagation patterns of the underlying generators of epileptic discharges.

## Methods and Materials

### EEG–MEG Inverse Problem Using Distributed Sources

The EEG–MEG inverse solution presented in this study uses a distributed source model where a large number of dipolar sources are distributed along the cortical surface. Considering the anatomical constraint that the orientation of each dipole is fixed perpendicular to the local cortical surface (Dale and Sereno 1993), the linear relationship between the source amplitude and the data is given by:

1 $${\mathbf{M}} = {\mathbf{GJ}} + {\mathbf{E}}$$

where **M** is the (*q* × τ) signal matrix acquired on *q* EEG or MEG channels at τ time samples. **E** models an additive measurement noise ((*q* × τ) matrix). **J** is a (*p* × τ) unknown matrix of the current intensity of the *p* dipolar sources along the tessellated cortical surface. **G** is the (*q* × *p*) lead field matrix obtained by solving the forward problem i.e., by estimating the contribution of each unit dipolar source on the sensors (Hallez et al. 2007).

### Maximum Entropy on the Mean (MEM) Framework

**Fig. 1:**
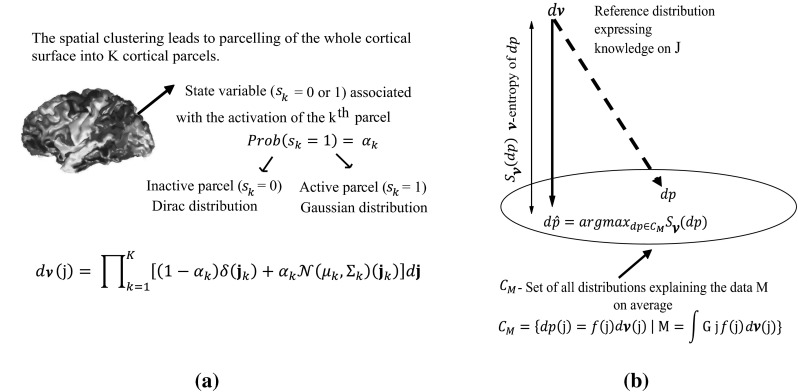
Maximum entropy on the mean (MEM) framework. **a** MEM initialization of the reference distribution $d\nu$: spatial clustering model that assumes brain activity to be organized into K cortical parcels showing homogenous activation state. This type of spatial clustering is obtained using data driven parcellization technique. After the definition of the state variable of the parcel, this *d*ν will be used to regularize the inverse problem. **b** MEM regularization algorithm: C_M_ represents the set of all the probability densities $dp$ that satisfy the data goodness of fit. Given the prior information on **J** in the form of reference distribution $d\nu$, the relative ν-entropy ($S_{\nu } (dp)$) measures the amount of information brought by the data **M**, with respect to the reference distribution $d\nu (j)$

To regularize the ill-posed inverse problem, the MEM framework incorporates prior information on **J** in the form of a reference distribution $d\nu (j)$. This reference distribution is a realistic spatial model that assumes brain activity to be organized into *K* (*K* ≪ *p*) cortical parcels showing homogenous activation states. This type of spatial clustering into *K* parcels (Fig. 1a) was obtained using a data driven parcellization (DDP) technique (Lapalme et al. 2006). To do so, first a projection method, namely the Multivariate Source Pre-localization (MSP) (Mattout et al. 2005) was applied to estimate a probability-like coefficient (MSP score) between 0 and 1 for each dipolar source on the cortical mesh, characterizing its contribution to the data. Then, using a region growing algorithm starting from the local optima of the MSP map, a parcellization of the full cortical surface into *K* non-overlapping parcels was estimated (see (Chowdhury et al. 2013) for further details).

Starting from this DDP, the reference distribution was modelled as follows:

2 $$d\nu (j) = \prod\limits_{k = 1}^{K} {\left[ {(1 - \alpha_{k} )\delta (j_{k} ) + \alpha_{k} N(\mu_{k} ,\varSigma_{k} )(j_{k} )} \right]dj}$$

Each cortical parcel *k*, assumed to be independent from the others, is characterized by an activation state $S_{k}$, describing if the parcel is active $(S_{k} = 1)$ or not $(S_{k} = 0)$. $\alpha_{k} = Prob(S_{k} = 1)$ is the probability of the *k*^*th*^ parcel to be active, which was initialized as the median of the MSP scores of the dipoles within the corresponding parcel. When the parcel is active $(S_{k} = 1)$, the dipole intensities within the *k*^*th*^ parcel are modeled using a Gaussian distribution $N(\mu_{k} ,\varSigma_{k} )$ where $\mu_{k}$ and $\varSigma_{k}$ represent respectively the mean and the covariance of the *p*_*k*_ dipoles within the *k*^*th*^ parcel. When the parcel is inactive $(S_{k} = 0)$, the dipole intensities are modeled using a Dirac distribution $\delta$, thus allowing to “shut down” the corresponding parcel.

Within the MEM framework, we consider the amplitude of the sources **J** to be estimated as a multivariate random variable described by a probability distribution $dp(j) = f(j)d\nu (j)$, where *f* is a ν-density of *dp*. Given the prior information on **J** in the form of reference distribution *d*ν, the relative ν-entropy ($S_{\nu } (dp)$) measures the amount of information brought by the data, with respect to the reference distribution $d\nu (j)$ (Amblard et al. 2004). Defining *C*_*M*_ as the set of probability measures on **J** that explains the data, ${\mathbf{M}} = \int {{\mathbf{G}}jf(j)d\nu (j)}$, on average (see Fig. 1b), the MEM solution consists in selecting $d\hat{p}$ that maximizes the ν-entropy and is the closest distribution to the reference distribution $d\nu$:

3 $$d\hat{p} = \arg \max_{{dp \in C_{M} }} S_{\nu } (dp)$$

under the constraints:${\mathbf{M}} - {\mathbf{G}}{\rm E}_{dp} [{\mathbf{J}}] = 0$ and $\int {dp(j) = 1}$, where ${\rm E}_{dp} [{\mathbf{J}}] = \int {jdp(j)}$. The MEM estimate of the source intensities ${\hat{\mathbf{J}}}$ is then found to be the expected value of the distribution $d\hat{p}$:

4 $${\hat{\mathbf{J}}} = {\rm E}_{{d\hat{p}}} [{\mathbf{J}}]$$

Such a regularization framework allows estimating the MEM solution through the optimization of a convex function within a *q* dimensional space, iteratively for each time sample. During the MEM optimization process, a noise covariance model is considered which is estimated as a diagonal matrix with a different value for each channel; thus taking into account the noise levels of each individual channel. For details on the MEM formulation, please refer to (Chowdhury et al. 2013).

In the present study, we will consider the coherent-MEM (cMEM) implementation, as described in (Chowdhury et al. 2013). In cMEM, additional constraint of local spatial smoothness in each parcel was introduced using diffusion-based spatial priors (Friston et al. 2008) in the initialization of the source covariance of every parcel ($\varSigma_{k}$). The mean intensity of every parcel ($\mu_{k}$) was initialized to zero. The spatial neighborhood order considered during the region growing procedure (cluster scale) has been fixed to a scale of 4, leading to approximately *K* = 200 parcels of size ≈2.5 cm^2^.

### Multimodal EEG–MEG Fusion Within the MEM Framework

**Fig. 2:**
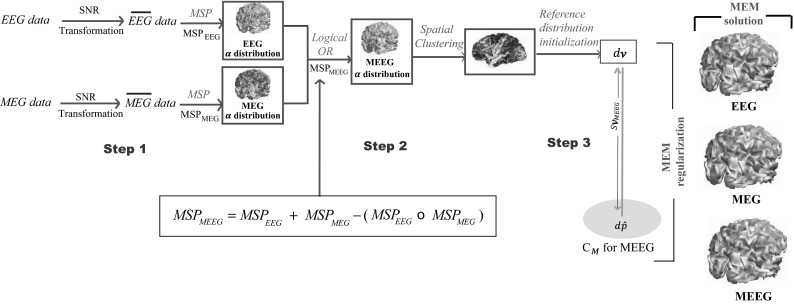
Multimodal EEG–MEG data fusion within the MEM framework. Step 1: normalization and concatenation of the data and lead field matrices from the two modalities. Step 2: parcellization of the cortical surface using the fusion of MSP scores (MSP_*MEEG*_). Step 3: initialization of the probability of activation of each parcel using MSP_*MEEG*_ and MEM regularization

The proposed EEG–MEG fusion within MEM framework consists of a 3-step fusion process, summarized in Fig. 2:

*Step 1Normalization and concatenation of the data and lead field matrices from the two modalities.* In order to integrate the two modalities, it is important to scale them to a common basis since they have different units and orders of magnitude. To do so, we applied a global mean signal to noise ratio (SNR) transformation of the data and the lead field, as described in (Fuchs et al. 1998) and (Ding and Yuan 2013). This SNR transformation consisted in estimating normalized dimensionless measures of EEG and MEG, using the mean standard deviation of some baseline data. Baseline data (${\mathbf{E}}_{EEG}$ and ${\mathbf{E}}_{MEG}$) consisted of real EEG and MEG background segments with the same duration (τ) as the data of interest **M** and exhibiting no epileptic discharges.

5 $$\sigma_{*} (i) = \sqrt {\frac{{\sum\limits_{t = 1}^{\tau } {\left( {E_{*} (i,t) - \bar{E}_{*} (i)} \right)^{2} } }}{\tau - 1}} \quad {\text{with}}\quad \bar{E}_{*} (i) = \frac{1}{\tau }\sum\limits_{t = 1}^{\tau } {E_{*} (i,t)}$$

where * refers to EEG or MEG, *i* is the index of the EEG or MEG channels, and *t* is the index of the τ time samples.

The mean standard deviation of the baseline over all sensors was then estimated as follows:

6 $$\bar{\sigma }_{*} = \frac{{\sum\limits_{i = 1}^{q} {(\sigma_{*} (i))} }}{{q_{*} }}$$

where $q_{*}$ is the number of EEG or MEG channels. The SNR transformation consisted in scaling the data and lead field matrices as follows:

7 $${\mathbf{M}}_{*}^{s} = {\raise0.7ex\hbox{${{\mathbf{M}}_{*} }$} \!\mathord{\left/ {\vphantom {{{\mathbf{M}}_{*} } {\bar{\sigma }_{*} }}}\right.\kern-0pt} \!\lower0.7ex\hbox{${\bar{\sigma }_{*} }$}}$$

8 $${\mathbf{G}}_{*}^{s} = {\raise0.7ex\hbox{${{\mathbf{G}}_{*} }$} \!\mathord{\left/ {\vphantom {{{\mathbf{G}}_{*} } {\bar{\sigma }_{*} }}}\right.\kern-0pt} \!\lower0.7ex\hbox{${\bar{\sigma }_{*} }$}}$$

Based on the scaled data and lead field matrices, the EEG-MEG fusion could be formalized using the following concatenation along the rows of the matrices (Fuchs et al. 1998; Henson et al. 2009; Ding and Yuan 2013):

9 $$\left[ {\begin{array}{*{20}c} {{\mathbf{M}}_{EEG}^{s} } \\ {{\mathbf{M}}_{MEG}^{s} } \\ \end{array} } \right] = \left[ {\begin{array}{*{20}c} {{\mathbf{G}}_{EEG}^{s} } \\ {{\mathbf{G}}_{MEG}^{s} } \\ \end{array} } \right]{\mathbf{J}} + \left[ {\begin{array}{*{20}c} {{\mathbf{E}}_{EEG}^{s} } \\ {{\mathbf{E}}_{MEG}^{s} } \\ \end{array} } \right]$$

where (${\mathbf{E}}_{EEG}^{s}$ and ${\mathbf{E}}_{MEG}^{s}$) refer to the scaled noise matrices. The symmetrical fusion of EEG and MEG will be further denoted by MEEG.

*Step 2Parcellization of the cortical surface using the fusion of MSP scores* (**MSP**_***MEEG***_). An originality of the MEM framework is to incorporate the complementary information provided by EEG and MEG through the reference distribution *d*ν. To do so, MSP scores were first computed from each modality separately (${\mathbf{MSP}}_{EEG}$ and ${\mathbf{MSP}}_{MEG}$), to assign for each modality a coefficient of activation of the sources. MSP was actually applied on a singular value decomposition of the scaled data:

10 $${\mathbf{M}}_{*}^{s} = {\mathbf{U}}_{*} {\mathbf{Y}}_{*} {\mathbf{V}}_{*}^{T} \quad {\text{where}}\quad * = {\text{EEG or MEG}}$$

where ${\mathbf{U}}_{*}$ is an orthogonal *q* × *q* matrix in which the *l*^*th*^ column vector is the sensor signature of the *l*^*th*^ component. ${\mathbf{V}}_{*}$ is an orthogonal τ × τ matrix, ${\mathbf{V}}_{*}^{\text{T} }$ denotes the transpose of ${\mathbf{V}}_{*}$. ${\mathbf{Y}}_{*}$ is an *q* × τ matrix whose diagonal contains the singular values of ${\mathbf{M}}_{*}^{s}$. With a selection of *l* functionally informed vectors ${\mathbf{U}}_{*}$, MSP scores were quantified by projecting the normalized lead field ${\overline{\mathbf{G}}_{*}}$ onto the normalized data ${\overline{\mathbf{U}}}_{*}$ (normalization by the norm of each column).

11 $${\mathbf{MSP}}_{*} = diag\left( {\overline{\mathbf{G}^{s}_{*}}^{T} \overline{{\mathbf{U}}} _{*} \overline{{\mathbf{U}}} _{*}^{T} \overline{{\mathbf{G}} _{*}^{s}} } \right),\quad {\text{where}}\quad * = {\text{EEG}}{\mkern 1mu}\,{\rm or}\, {\mkern 1mu} {\text{MEG}}$$

With such a projection **MSP**_*EEG*_ or **MSP**_*MEG*_ scores estimated a probability-like coefficient assessing the contribution of each dipolar source to the corresponding EEG and MEG data. A second level of EEG/MEG fusion was then introduced, using a logical OR operation (∨) on **MSP**_*EEG*_ and **MSP**_*MEG*_ scores, in order to taken into account the contribution of the dipolar sources either to EEG or MEG or both data.

12 $${\mathbf{MSP}}_{MEEG} = {\mathbf{MSP}}_{EEG} \vee {\mathbf{MSP}}_{MEG} = {\mathbf{MSP}}_{EEG} + {\mathbf{MSP}}_{MEG} - ({\mathbf{MSP}}_{EEG} \circ {\mathbf{MSP}}_{MEG} )$$

where ° denotes the Schur (Hadamard) product of the two matrices leading to element-wise multiplication of their elements. DDP was then applied using these fused MSP scores (${\mathbf{MSP}}_{MEEG}$) in order to obtain parcellization of the full cortical surface driven by information provided by MEEG fusion data.

*Step 3Initialization of the probability of activation of each parcel*$\alpha_{k}$ using ${\mathbf{MSP}}_{MEEG}$. Given the parcellization obtained in **Step 2**, we then considered a 3rd level of the EEG/MEG fusion by using the median of the fused MSP scores (${\mathbf{MSP}}_{MEEG}$) within the *k*^*th*^ parcel to initialize $\alpha_{k}$ i.e., the probability of each parcel to be active (cf. “Maximum entropy on the mean (MEM) framework” section, Eq. 2).

This three-level fusion scheme was proposed to integrate the complementary information provided by both modalities within the MEM framework. Then starting from the initialized reference model *dν* estimated from fused MEEG data, MEM regularization was used to find a solution from SNR-transformed concatenated MEEG data, as illustrated in Fig. 2.

### Minimum Norm Estimate and Other Variants with L-Curve Method

In the present study, we will compare the performance of cMEM with MNE method and two noise-normalized variants of MNE—dynamic statistical parametric mapping (dSPM) (Dale et al. 2000) and standardized low-resolution electromagnetic tomography (sLORETA) (Pascual-Marqui 2002).

(a) MNE: With the assumption that all sources are independent and have same energy, MNE solution (${\hat{\mathbf{J}}}_{{{\mathbf{mne}}}}$) provides the minimum energy of the current distribution **J** (Dale and Sereno 1993; Hämäläinen and Ilmoniemi 1994). The L-curve method (Hansen 2000) was used to estimate the regularization hyper-parameter (λ), allowing the best balance between data fit ($\left\| {{\mathbf{M - GJ}}} \right\|^{2}$) and the a priori constraint ($\left\| {\mathbf{J}} \right\|^{2}$), within the following optimization scheme:

13 $$\begin{aligned} {\hat{\mathbf{J}}}_{mne} &= \arg\min_{J} \left( \left\| {\mathbf{M}} - {\mathbf{GJ}} \right\|^{2} + \lambda \left\| {\mathbf{J}} \right\|^{2} \right) \\ &= \left({\tilde{\mathbf{G}}}^{\mathbf{T}} {\varvec{\Sigma}}_{\mathbf{d}} {\tilde{\mathbf{G}}} + \lambda {\varvec{\Sigma}}_{\mathbf{s}} \right)^{ - 1} {\tilde{\mathbf{G}}}^{\mathbf{T}} {\varvec{\Sigma}}_{\mathbf{d}} {\tilde{\mathbf{M}}} = {\tilde{\mathbf{W}}}_{\mathbf{MNE}}{\tilde{\mathbf{M}}} \\ \end{aligned}$$

where, ${\tilde{\mathbf{M}}} = {\varvec{\Sigma}}_{{\mathbf{d}}}^{{{\mathbf{ - 1/2}}}} {\mathbf{M}}$ and ${\tilde{\mathbf{G}}} = {\varvec{\Sigma}}_{{\mathbf{d}}}^{{{\mathbf{ - 1/2}}}} {\mathbf{G}}$ are the spatially whitened data and gain matrices, respectively.${\tilde{\mathbf{W}}}_{{{\mathbf{MNE}}}}$ is the classical MNE inverse operator with ${\varvec{\Sigma}}_{{\mathbf{s}}}$ as the identity source covariance matrix and ${\varvec{\Sigma}}_{{\mathbf{d}}}$ as the diagonal noise covariance matrix of the whitened data resulting in an identity matrix. In order to evaluate EEG/MEG fusion using MNE, data were normalized as in Eqs. (7) and (8), spatially pre-whitened and concatenated as in Eq. (9), and MNE was then directly applied to concatenated matrices.

Both dSPM and sLORETA are derived from ${\tilde{\mathbf{W}}}_{{{\mathbf{MNE}}}}$  by normalizing the rows of the inverse operator.

(b) dSPM (Dale et al. 2000): The estimated current at each source location is divided by an estimate of the noise at that location, which can be obtained by applying ${\tilde{\mathbf{W}}}_{{{\mathbf{MNE}}}}$ to the signal covariance matrix as follows:

14 $$\begin{aligned} {\tilde{\mathbf{W}}}_{{{\mathbf{dSPM}}}} = \left( {\sqrt {diag({\tilde{\mathbf{W}}}_{{{\mathbf{MNE}}}} \varvec{\Sigma}_{{\mathbf{d}}} {\tilde{\mathbf{W}}}_{{{\mathbf{MNE}}}}^{{\mathbf{T}}} )} } \right){\tilde{\mathbf{W}}}_{{{\mathbf{MNE}}}} \hfill \\ {\mathbf{J}}_{{{\mathbf{dSPM}}}} ={\tilde{\mathbf{W}}}_{{{\mathbf{dSPM}}}} {\mathbf{M}} \hfill \\ \end{aligned}$$

(c) sLORETA (Pascual-Marqui 2002): consists in a similar approach, but the normalization is obtained from the variance of the estimated sources, instead of using just the variance due to the noise component.

15 $$\begin{aligned} {\tilde{\mathbf{W}}}_{{{\mathbf{sLORETA}}}} = \left( {\sqrt {diag({\tilde{\mathbf{W}}}_{{{\mathbf{MNE}}}} {\mathbf{(GG}}^{{\mathbf{T}}} + {{\varvec{\Sigma}}}_{{\mathbf{d}}}) {{{\tilde{\mathbf{W}}}}}_{{{\mathbf{MNE}}}}^{{\mathbf{T}}} )}} \right){\tilde{\mathbf{W}}}_{{{\mathbf{MNE}}}} \hfill \\ {\mathbf{J}}_{{{\mathbf{sLORETA}}}} = {\tilde{\mathbf{W}}}_{{{\mathbf{sLORETA}}}} {\mathbf{M}} \hfill \\ \end{aligned}$$

whereas MNE localization is biased towards more superficial sources, dSPM and sLORETA actually implicitly perform some “depth weighting” because of the noise normalization—sources with generally higher amplitude will be normalized by higher noise levels or source variances (Hauk et al. 2011).

### Evaluation Procedure

The proposed MEM fusion approach was evaluated in a well-controlled environment using realistic simulations of EEG and MEG inter-ictal epileptic spikes. The geometry and the anatomy of our simulation environment were derived from a real patient’s dataset.

#### Realistic Simulations

*Geometry dataset* Simultaneous EEG/MEG acquisition was performed on a patient with focal epilepsy using a 275 channel CTF-MEG system (272 active sensors) and a 54 channel EEG-cap (Easy-cap, Herrsching, Germany) at a sampling rate of 1200 Hz. The 54 EEG electrodes were placed according to the 10–20 system with additional electrodes according to the 10–10 system especially covering the inferior temporal and parietal regions (FT9, P9, FT10, and P10). Written informed consent for this study was obtained from the patient. EEG and MEG data containing no traces of IEDs were recorded from this patient, which was used in the simulation model to create realistic noise.

*Anatomy dataset* A high resolution T1-weighted anatomical MRI of the same patient was used to segment the surfaces of the brain to obtain a realistic head model. The distributed source model was obtained by segmenting the grey-white matter interface from the MRI using BrainVISA-4.2.1 software^1^ (Mangin et al. 1995). The source model consisted in a realistic 3D mesh of the cortical surface (8000 vertices, 4 mm resolution). Using the OpenMEEG (Gramfort et al. 2011) implementation in Brainstorm software (Tadel et al. 2011), we generated a 3-layer EEG boundary element method (BEM) model consisting of the inner skull, outer skull and the scalp (conductivity values of 0.33:0.0165:0.33 S/m) and a 1-layer MEG BEM model consisting of the inner skull (conductivity value of 0.33 S/m).

##### Static Simulation Model

These simulations were similar to the ones considered in (Chowdhury et al. 2013). 100 simulation configurations involving one spatially extended source exhibiting spiking activity were randomly generated on the cortical mesh. The position of each source was selected by choosing a seed point randomly on the cortical surface mesh. The spatial extent of each source was obtained by region growing around the seed following the cortical surface using spatial neighborhood order *s*_*e*_ = 3 (≈4 cm^2^) and *s*_*e*_ = 4 (≈12 cm^2^). The time course of the simulated sources was the time course of an epileptic spike modeled with three Gamma functions, although only signal around the main peak of the spike was analyzed. Let us refer ${\mathbf{Jth}}$ as the simulated theoretical current distribution obtained from the spatial distribution of the simulated sources together with the corresponding time course. EEG and MEG data were then simulated by applying the forward model ${\mathbf{G}}_{{{\mathbf{EEG}}}}$ and ${\mathbf{G}}_{{{\mathbf{MEG}}}}$ to the simulated current density, respectively. Realistic physiological noise was extracted from a 3 min segment of EEG/MEG background activity acquired on the selected patient and added to the simulated data. The amplitude of the background activity trials was scaled to ensure a signal-to-background ratio of 1 (0 dB) for most superficial sources when using reference source amplitude of 9.5 nA m for each dipolar source along a patch of 6 cm^2^. Consequently, the SNR of the realistic simulated data varied depending upon the location and extent of the underlying sources. In this set of 100 simulations, the SNR ranged approximately between 1 and 12. Note that as opposed to our previous study (Chowdhury et al. 2013), here only 1 trial of background EEG/MEG data was used in the simulations, thus mimicking the occurrence of single non-averaged spikes.

We considered the following indicators to characterize the simulations:

1. *Eccentricity*—Eccentricity is defined as the mean Euclidean distance between all vertices of the simulated patch and the center of the head model.^2^ Most superficial sources had an eccentricity value higher than 80 mm. Sources with eccentricity ranging between 60 and 80 mm corresponded mainly to mesio-temporal sources and the ones with eccentricity lower than 60 mm corresponded to the sub-cortical sources.

2. *Cancellation index*—This index estimates the amount of overlap between signal patterns of individual sources within an active patch leading to signal cancellation (notably caused by dipolar sources oriented in opposite directions on both walls of a sulcus), as proposed by (Ahlfors et al. 2009).

16 $$Ic = 1 - \frac{{\sqrt {\sum\limits_{l \in N} {\left( {\sum\limits_{i = 1}^{q} {G(i,l)} } \right)^{2} } } }}{{\sum\limits_{i = 1}^{q} {\sqrt {\sum\limits_{l \in N} {G^{2} (i,l)} } } }}$$

where *i* is the index of summation over all *q* sensors, *l* is the index of summation over all elements in the set of *N* active dipoles located within the simulated patch. *G*(*i*,*l*) is the value of the *i*th row and *l*th column of the lead field matrix **G**. This index ranges between 0 and 1, *Ic* = 1 indicates full cancellation and *Ic* = 0 indicates no cancellation effect.

##### Spatio-temporal Simulation Model

Hundred simulation configurations were randomly generated on the cortical mesh, involving activation of two spatially extended sources following the same time course but presenting a 15 ms delay between them. These simulations were proposed to mimic axonal propagation between two distant spike generators, with significant overlap between the time courses of the two generators. The sources were spatially separated by a fixed geodesic distance of 73 mm (i.e., a spatial neighborhood order of 10) and both sources were located in the same hemisphere. The velocity of this simulation model mimics the velocity of real propagating spikes (varying from 1 to 40 m/s) (Emerson et al. 1995). This type of propagation is concordant with literature and can express a remote activation of a neural network connected to an active population by a fiber tract (Baumgartner et al. 1995; Huppertz et al. 2001). For this set of 100 simulations, the spatial neighborhood order was *s*_*e*_ = 3 consisting of sources with spatial extent ranging from 2 to 6 cm^2^. One trial of real background was added on noise-free simulated data. The amplitude of the background activity trials was scaled to ensure a larger signal-to-background ratio (3≈4.7 dB) than the static simulations as the spatio-temporal simulations involve more complex source patterns to recover. Consequently the SNR for this set of propagating spikes ranged approximately between 2 and 9.

##### Impact of the Number of EEG Electrodes Considered During MEEG Fusion

The static simulation model was considered to generate EEG and MEG data, while the impact of three different EEG configurations derived from the 10–10 electrode placement system was evaluated: A complete EEG setup involving 54 EEG electrodes (see Fig. 7a EEG topography for the 54 EEG electrodes set-up), and two down-sampled montages involving respectively 32 and 20 EEG electrodes (see Fig. 9a EEG topographies for the two down sampled EEG electrodes set-up). Note that the 20 EEG electrodes set-up was similar to the conventional 10-20 EEG system used in most clinical centers.

##### Impact of Model-Error

We are aware that the use of same head model during forward and inverse problem can lead to the best case scenario in any simulation study. In order to mimic real data scenario, one can introduce noise in the measurement through mis-modelling in simulations (Wang and Ren 2013). We evaluated the robustness of cMEM method by varying the tissue conductivities in the EEG forward model during EEG and MEEG source localization. The correct modeling of head tissue conductivities, especially the conductivity ratio of the skull relative to brain and scalp is an important parameter that determines the accuracy of the forward and inverse solution especially in EEG. In the literature (Oostendorp and Delbeke 1999; Lai et al. 2005; Zhang et al. 2006; Lew et al. 2009), similar conductivity values for the brain and scalp (ranging from 0.12 to 0.48 S/m) have been reported. However, estimation of the skull conductivity has been reported to be more inconsistent with values ranging between 0.006 and 0.080 S/m (Hoekema et al. 2003). We extrapolated from past studies (Oostendorp and Delbeke 1999; Malmivuo and Suihko 2001; Lai et al. 2005; Zhang et al. 2006; Huiskamp 2008; Vallaghé and Clerc 2009; Fangmin Chen 2010) a range of brain-to-skull conductivity ratio (that will be denoted Rbs) to be tested: Rbs ranging between 1:15 and 1:25 was found acceptable for the adult brain. For this test, we performed two sets of simulations. In the first set, we simulated EEG signals using different Rbs (randomized between 1:15 and 1:25 following a normal distribution with mean 1:20 and standard deviation of 1:3.3) of the EEG head model for 50 randomly placed sources and localized these sources using EEG head model at one Rbs (1:20). In the second set, we considered the same Rbs of 1:20 for both simulation and localization over the same 50 sources as the first set. Then we compared the localization accuracy (AUC) of cMEM on the two set of simulations for EEG and MEEG data.

##### Validation Metrics

As the Ground Truth was fully controlled using simulated data, we considered the following validation metrics to evaluate the performances of MNE and cMEM source localization methods when applied on EEG, MEG or MEEG data. Some of the metrics have been described in further details in our previous studies, (Chowdhury et al. 2013) and (Grova et al. 2006).

1. Area Under the Receiver Operating Characteristic (ROC) curve, AUC—was used to assess the detection ability of the localization methods. The AUC index looks at the normalized energy of each source at a specific time sample. In case of static simulations, the energy at the main peak (τ_0_) of the simulated spike was considered. For the 2-source spatio-temporal simulations, the AUC index was estimated separately at the peak of each source spike while removing the contribution of the vertices of the second source. Since the spatio-temporal simulation involved activation of two sources separated by a temporal delay of 15 ms (with some temporal overlap), it was possible to estimate AUC for each source separately at the time of their peak.

This detection accuracy index (between 0 and 1) integrates sensitivity and specificity of the source localization methods to reconstruct the spatial extent of the source against the Ground Truth, by varying a detection threshold between 0 and the maximum of reconstructed current density. More details on AUC estimation can be found in Appendix. An AUC value greater than 0.8 was considered good detection accuracy.

2. Spatial dispersion (SD)—proposed in (Molins et al. 2008), measures both the spatial spread of the estimated source distribution around the true source location and the localization error between the estimated source distribution and the true source location. Let us denote by ${\hat{\mathbf{J}}}$ the result of the source localization method to be evaluated. Then, $\hat{J}(i,\tau_{0} )$ represents the amplitude of the current density distribution estimated for a dipolar source *i* on the cortical surface at the main peak of IED (τ_0_). To measure the SD of this solution, we weight the amplitude of all the *p* cortical sources by their minimum distances from the simulated patch using the following formula:

17 $$SD(j) = \sqrt {\frac{{\sum\limits_{i = 1}^{p} {\left( {\min_{j \in \varTheta } (D(i,j))\hat{J}^{2} (i,\tau_{0} )} \right)} }}{{\sum\limits_{i = 1}^{p} {\hat{J}^{2} (i,\tau_{0} )} }}}$$

where $\min_{j \in \varTheta } (D(i,j))$ provides the minimum Euclidean distance between the source *i* and the sources *j* in the simulated patch. $\varTheta$ denotes the set of indices of the dipoles in the simulated patch and this minimum distance is zero when the source *i* belongs to $\varTheta$. SD values close to zero means there is no active source outside the simulated patch. Large SD values could be caused either by the presence of sources far away from the true source that are contributing to the estimated solution (spurious sources) or by the spatial spread of the reconstructed source around the true extent of the simulated patch.

3. Shape error (SE)—In order to assess the accuracy of the reconstructed time courses within the simulated patch, we proposed the metric SE as the root mean square of the difference between the normalized theoretical source distribution (${\mathbf{Jth}}$) and the normalized estimated source distribution (${\hat{\mathbf{J}}}$). Therefore, SE for a simulated source was estimated as follows:

Let us consider $Jth(i,t)$ and $\hat{J}(i,t)$, where $i \in \varTheta$ and *t* is the time parameter.

18 $$SE = \sqrt {\tfrac{1}{\tau }\sum\limits_{t}^{\tau } {\left( {\tfrac{m(Jth(t))}{{\max_{t} \left( {\left| {m(Jth(t)} \right|} \right)}} - \tfrac{{m(\hat{J}(t))}}{{\max_{t} \left( {\left| {m(\hat{J}(t))} \right|} \right)}}} \right)}^{2} }$$

with $m(Jth(t)) = \frac{1}{\varTheta }\sum\limits_{i \in \varTheta } {Jth_{n} (i,t)}$ and $m(\hat{J}(t)) = \frac{1}{\varTheta }\sum\limits_{i \in \varTheta } {\hat{J}_{n} (i,t)}$. The subscript “n” in $Jth_{n}$ or $\hat{J}_{n}$ denotes the normalization of the matrix $\hat{J}$ so that its values are between -1 and 1, for example:$J_{n} (i,t) = \frac{{\left| {J(i,t)} \right|}}{{\max_{j} \left( {\left| {J(i,t)} \right|} \right)}}$. $\max_{t}$ is the maximum over *t* time samples.

#### Application of MEM Fusion on Clinical Data

We evaluated our proposed MEEG fusion method on clinical data acquired from two patients with intractable focal epilepsy. We selected IEDs that occurred simultaneously in both EEG and MEG signals, while making sure that the individual IED on either EEG or MEG had high SNR (at least SNR of 1). SNR was estimated as the ratio between the maximum signal measured at the peak of the spike (over all channels) and the standard deviation of some baseline data (2 s of data showing normal traces with no epileptic activity). We also carefully checked that the selected IEDs exhibited similar topographic maps.

Patient 1 is suffering from a cryptogenic focal epilepsy with a left fronto-temporal epileptic focus (defined by EEG telemetry and seizure semiology). In Patient 2 a Focal Cortical Dysplasia (FCD) was diagnosed based on the MRI in the left frontal opercular region. These patients participated as research subjects of the project entitled: “Application of magnetoencephalography in the assessment of the epileptic focus” (Dr. E. Kobayashi being the principal investigator for this project). Written informed consent for this study was obtained from the patients.

Analysis of the IEDs involved:

1. Data acquisition—simultaneous EEG/MEG recordings were acquired using a 275 channel CTF-MEG-system using a 54 channel EEG-cap. EEG electrodes were placed according to the 10/20 system, with additional electrodes according to the 10/10 system covering the inferior temporal and parietal regions. EEG/MEG signals were recorded with patients at rest in a supine position. No filters were applied to the MEG recording and a hardware high pass filter of 0.03 Hz was used for the EEG. The sampling rate was 2400 Hz.

2. Pre-processing of EEG/MEG data—standard CTF software was used to process the data offline. Data were down-sampled to 600 Hz and DC-offset was removed. Filtering included 0.3–70 Hz bandpass filter (butterworth, 4th order) and 60 Hz notch filter (and its harmonics). Any bad channels were removed.

3. Visual analysis and marking of EEG/MEG data—IEDs were visually marked by a clinical neurophysiologist (MH). Only simultaneous EEG and MEG spikes were analyzed.

4. Pre-processing of image data—preprocessing of MRI data, co-registration and forward model estimation were done similarly to the simulated data in “Realistic simulations**”** section Anatomy dataset.

5. Solving the inverse problem—we performed single spike localization of EEG, MEG and MEEG data using cMEM.

Single spike source localization was performed within a time window of 700 ms around the peak of the marked spike (200 ms before and 500 ms after). For each single spike, we identified (based on the SNR level), the first significant MEG peak and the first significant EEG peak, since these two peaks were not always synchronous.

## Results

### Performance of Fusion Approach on Static Simulation

**Fig. 3:**
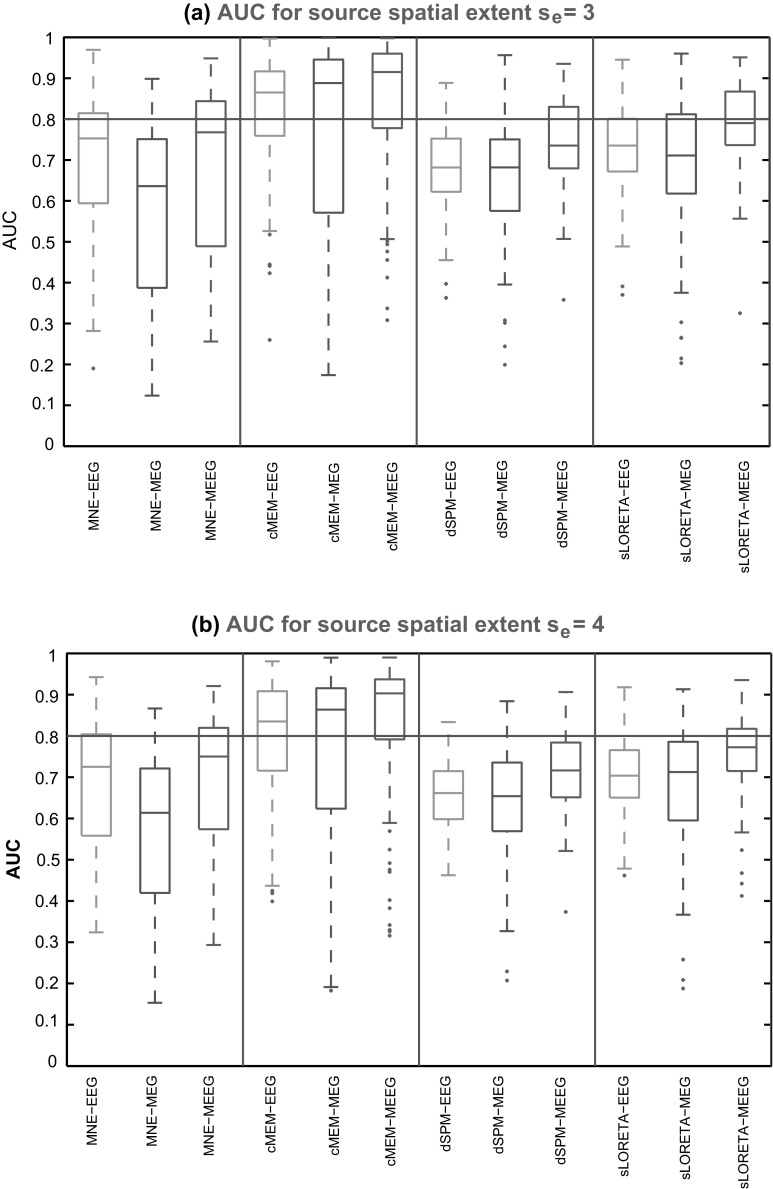
Distribution of AUC results over 100 simulations of randomly placed single static source for source localization methods, MNE, cMEM, dSPM and sLORETA on the three modalities (EEG, MEG and MEEG). **a** *Boxplot* representation of AUC values for simulated sources with spatial extent *s*_*e*_ = 3, **b** *Boxplot* representation of AUC values for simulated sources with spatial extent *s*_*e*_ = 4. (*Horizontal line*, AUC = 0.8). Color code for each modality: EEG in *green*, MEG in *blue* and MEEG in *red* (Color figure online)

**Fig. 4:**
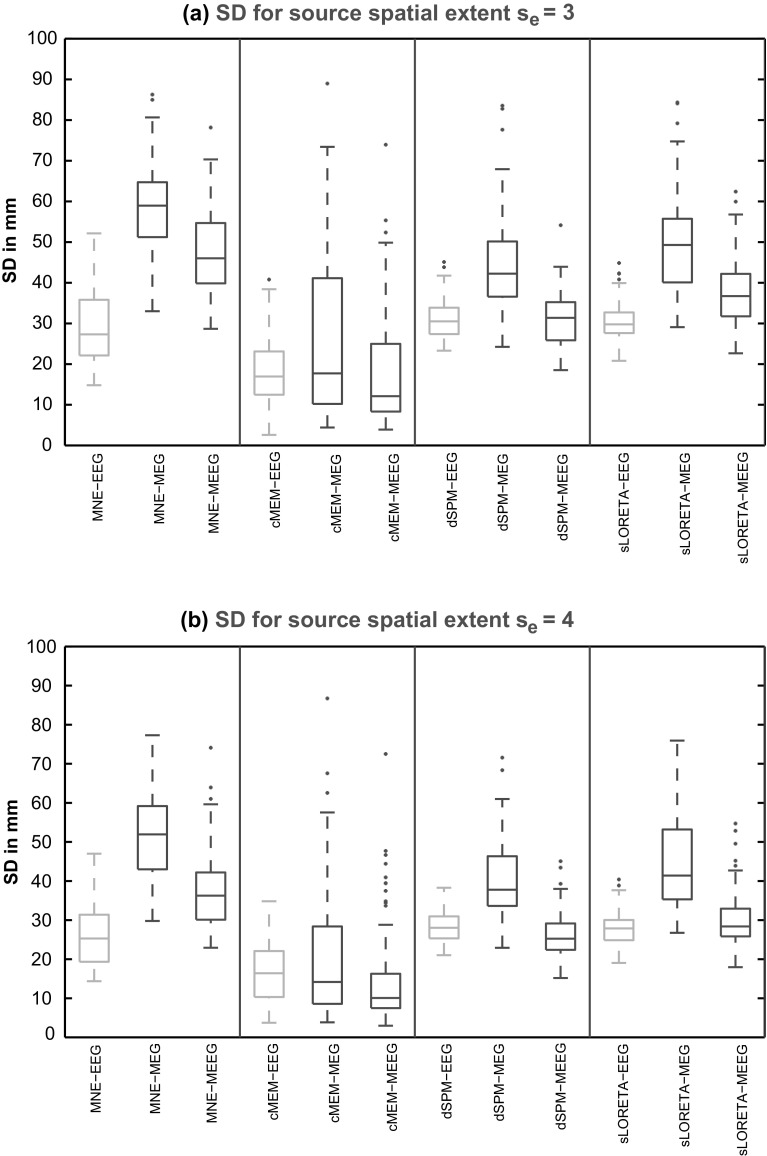
Distribution of SD results over 100 simulations of randomly placed single static source for source localization methods, MNE, cMEM, dSPM and sLORETA on the three modalities (EEG, MEG and MEEG). **a** *Boxplot* representation of SD values (in mm) for simulated sources with spatial extent *s*_*e*_ = 3. **b** *Boxplot* representation of SD values (in mm) for simulated sources with spatial extent *s*_*e*_ = 4. Color code for each modality: EEG in *green*, MEG in *blue* and MEEG in *red* (Color figure online)

We observed an overall good detection accuracy for cMEM on all modalities (median AUC >0.8) for sources with spatial extents *s*_*e*_ = 3 and 4 (Fig. 3a, b). Similarly to our previous findings in (Grova et al. 2006) and (Chowdhury et al. 2013), MNE was less sensitive than cMEM to the spatial extent of the sources, showing overall lower AUC values. For the first time, we also clearly demonstrated that cMEM performed better than dSPM and sLORETA when recovering the spatial extent of the underlying generators. Notice the better performance for all the methods when using MEEG, as opposed to EEG or MEG alone. The validation metric SD exhibited clearly lower values for cMEM when compared to MNE, dSPM and sLORETA (Fig. 4), suggesting less spatial spread around the true source and/or less distant spurious sources. From Fig. 4a and b, we observed that for all the methods the median of SD distribution for MEG was larger than for EEG and MEEG suggesting the presence of more spurious sources mis-localized outside the active region for MEG. The shape of the distribution for SD values when using MEG had long tails towards larger values. We checked that this was caused by misleading reconstructions for simulated mesial or deep generators. Interestingly, for all the methods, SD values for MEEG were the lowest indicating a more accurate estimation of the spatial extent of the generators and less spurious sources outside the simulated region, when compared to EEG and MEG localizations.

**Fig. 5:**
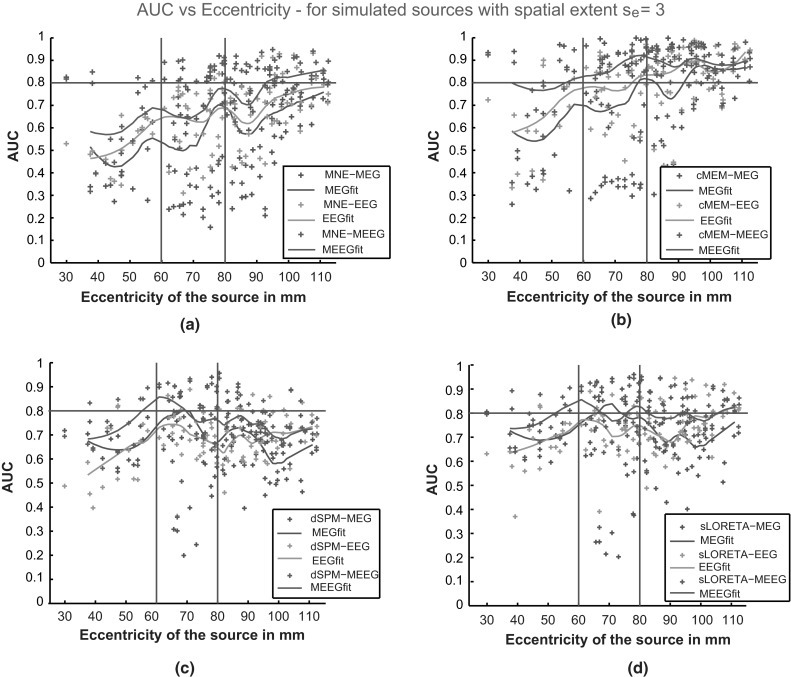
AUC as a function of eccentricity of the sources for 100 simulations involving randomly placed single static source at different locations for source localization methods MNE, cMEM, dSPM and sLORETA on the three modalities (EEG, MEG and MEEG). **a** AUC values obtained for MNE, **b** for cMEM, **c** for dSPM, and **d** for sLORETA when localizing simulated sources with spatial extent *s*_*e*_ = 3. *Solid lines* are the moving average of the AUC values for the respective methods. *Horizontal line*, AUC = 0.8, *vertical lines*: eccentricity = 60 and 80 mm. Color code for each modalities: EEG in *green*, MEG in *blue* and MEEG in *red* (Color figure online)

**Fig. 6:**
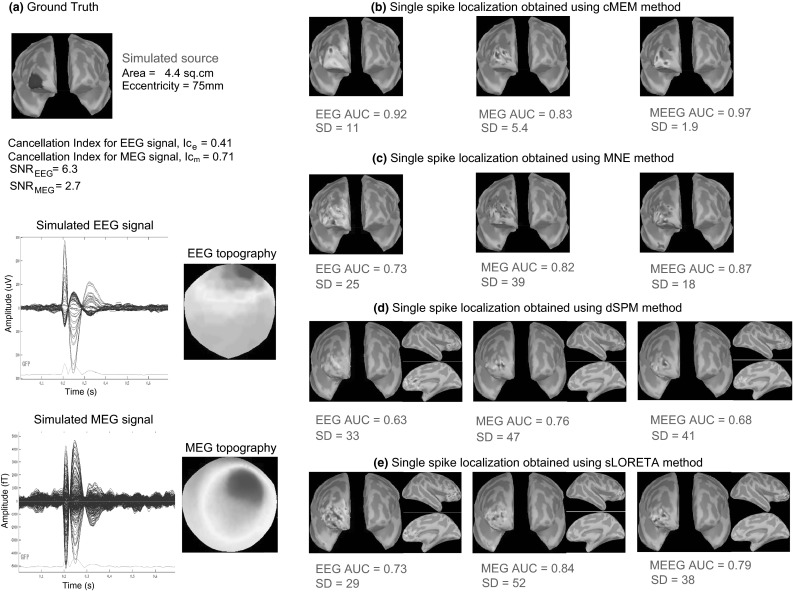
Qualitative assessment for example of static simulation. Visual analysis of source localization results together with AUC and SD values for a single static simulated source with area = 4.4 cm^2^ and eccentricity 75 mm. All source localization results are presented as the absolute value of the current density at the peak of the spike, normalized to its maximum activity and thresholded upon the level of background activity. **a** Theoretical simulated source: area and eccentricity of the cortical source; associated simulated EEG and MEG signal and topography for all 54 EEG and 272 MEG channels respectively; Cancellation index for the simulated source in EEG, *Ic*_*e*_ = 0.41 and in MEG, *Ic*_*m*_ = 0.71; SNR for EEG signal, *SNR*_*EEG*_ = 6.3 and for MEG signal, *SNR*_*MEG*_ = 2.7. **b** Source localization results obtained using cMEM on EEG, MEG and MEEG data. **c** Source localization results obtained using MNE on EEG, MEG and MEEG data. **d** Source localization results obtained using dSPM on EEG, MEG and MEEG data. **e** Source localization results obtained using sLORETA on EEG, MEG and MEEG data

The behavior of AUC as a function of the eccentricity of the simulated sources is presented in Fig. 5. As expected, for all the three modalities, we noticed better localization for superficial sources (eccentricity >80 mm, AUC >0.8 for cMEM) than for mesial and deeper sources (eccentricity <60 mm) for MNE and cMEM. EEG performed slightly better than MEG for most mesial sources (60 mm < eccentricity < 80 mm). However, dSPM and sLORETA provided similar localization accuracy for sources at all eccentricities; thus confirming that these methods are indeed less biased towards superficial sources. MEEG improved the detection accuracy of the methods for sources at all eccentricities. Overall, cMEM on MEEG data proved to be the most accurate (AUC >0.8) method showing good spatial accuracy for most sources, mainly superficial but also for some deeper ones. We also checked that the largest SD values in Fig. 4a and b were mainly due to mis-localized deep sources with low eccentricity (results not shown). As a particular example, Fig. 6 illustrates the ability of cMEM, MNE, dSPM, and sLORETA to localize a right superior frontal simulated source using EEG, MEG and MEEG data. Source localization results are presented over the inflated cortical surface, using Brainstorm software (Tadel et al. 2011). AUC and SD values were in agreement with visual inspection. We observed the largest AUC values (0.97) and smallest SD value (1.9) for cMEM when localizing MEEG data (Fig. 6b). This result along with the findings from Figs. 3 and 4 suggests that MEEG localization using cMEM was the most accurate method in detecting the spatial extent of the source. SD for MNE was very large, especially for EEG and MEG localizations whereas for dSPM and sLORETA, SD was very large for all the three modalities. This corroborates with the visual analysis, showing an overestimation of the spatial extent and the presence of several spurious sources located far from the active region (in fronto-mesial and temporal regions notably), whereas the maximum of reconstructed activity was indeed accurately estimated. Overall, for all the methods, we noticed an improvement in spatial accuracy when localizing MEEG data, when compared to monomodal EEG and MEG localizations.

**Fig. 7:**
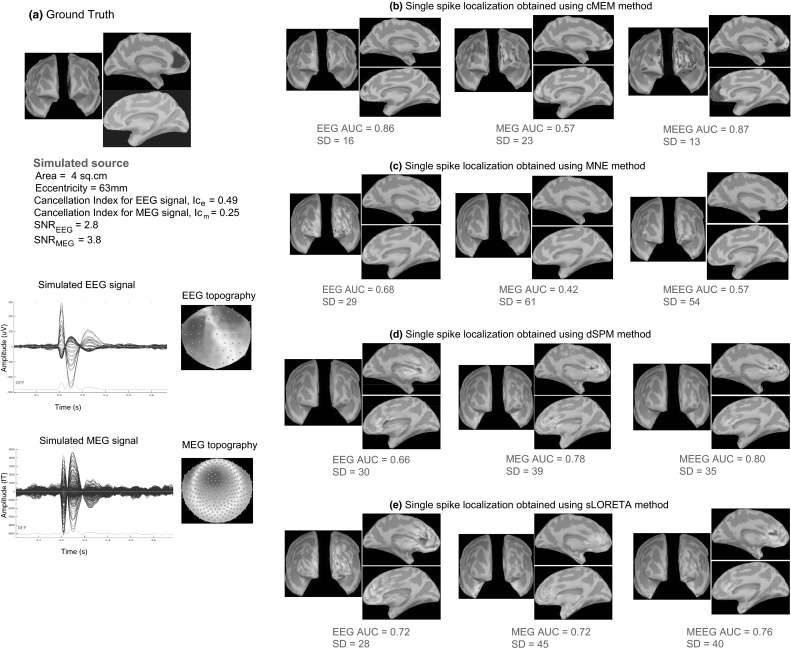
Qualitative assessment for an example of static simulation. Visual analysis of source localization results together with AUC and SD values for a single static simulated source with area = 4 cm^2^ and eccentricity 63 mm. All source localization results are presented as the absolute value of the current density at the peak of the spike, normalized to its maximum activity and thresholded upon the level of background activity. **a** Theoretical simulated source: area and eccentricity of the cortical source; associated EEG and MEG topography; Cancellation index for the simulated source in EEG, *Ic*_*e*_ = 0.49 and in MEG, *Ic*_*m*_ = 0.25; SNR for EEG signal, *SNR*_*EEG*_ = 2.8 and for MEG signal, *SNR*_*MEG*_ = 3.8. **b** Source localization results obtained using cMEM on EEG, MEG and MEEG data. **c** Source localization results obtained using MNE on EEG, MEG and MEEG data. **d** Source localization results obtained using dSPM on EEG, MEG and MEEG data. **e** Source localization results obtained using sLORETA on EEG, MEG and MEEG data

Figure 7 illustrates the localization of a left deep cingulate simulated source with cMEM, MNE, dSPM, and sLORETA when considering EEG, MEG and MEEG data. Overall, for all the methods, AUC and SD values showed that MEEG improved the localization, especially since fusion lead to higher AUC values and lower SD values than when considering MEG and EEG alone. MEEG localization using cMEM involved sources well localized on the left hemisphere, but with larger amplitudes towards the more superficial and fronto-polar vicinity of the generator. As expected, due to the implicit depth-weighting behavior of dSPM and sLORETA, these methods were able to recover the deeper aspects of the source (anterior cingulate sulcus) more accurately than cMEM or MNE. However, despite the fact that the main generator was found, both sLORETA and dSPM presented also spurious sources in the deeper regions of both hemispheres (including posterior cingulate gyrus and thalamus), resulting in misleading evaluation (i.e., high SD values and low AUC values). We noticed these spurious deep sources even in the previous example involving just a superficial source (Fig. 6d, e).

### Impact of the Number of EEG Electrodes Considered During MEEG Fusion

**Fig. 8:**
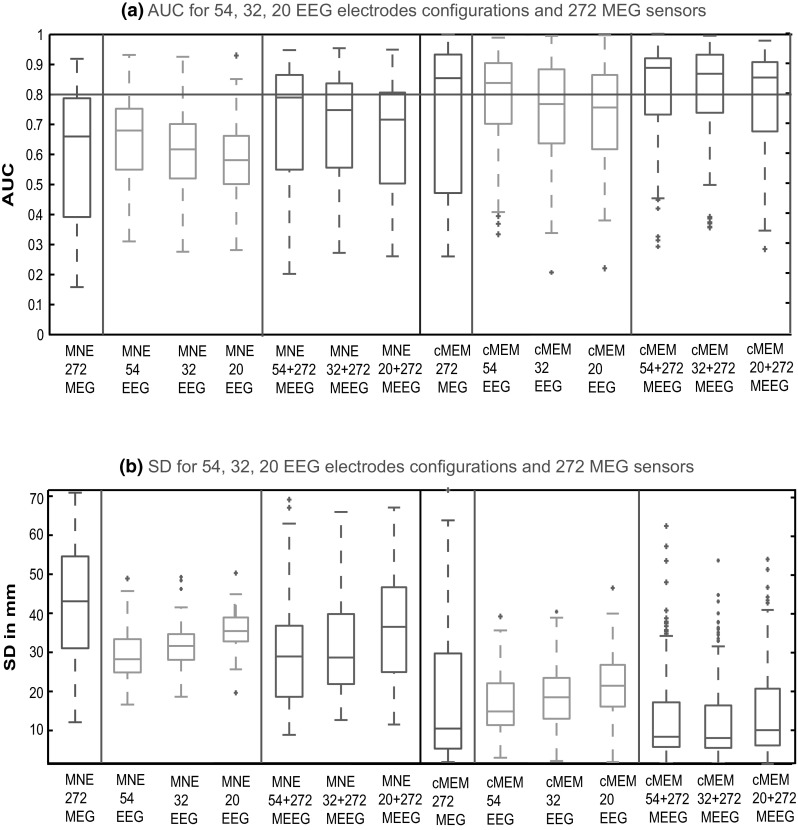
Evaluation of the source localization methods for three configurations of EEG electrodes using the detection accuracy index AUC and SD values. **a** Distribution of AUC values using* boxplot* representation over 100 simulated sources with spatial extent *s*_*e*_ = 3 for MNE and cMEM methods applied on: (from *left* to *right*) 272 MEG sensors in *blue*, 54, 32, and 20 EEG channels in red and 272 MEG + 54 EEG, 272 MEG + 32 EEG, 272 MEG + 20 EEG channels in *red*. **b** Distribution of SD values using* boxplot* representation over 100 simulated sources with spatial extent *s*_*e*_ = 3 for MNE and cMEM methods applied on: (from *left* to *right*) 272 MEG sensors in *blue*, 54, 32, and 20 EEG channels in *red* and 272 MEG + 54 EEG, 272 MEG + 32 EEG, 272 MEG + 20 EEG channels in *red* (Color figure online)

Figure 8a presents the distribution of AUC values obtained on 100 static simulations, when decreasing the number of EEG electrodes. As expected, we observed a decrease of AUC for EEG source localization when reducing the number of EEG electrodes, for both MNE and cMEM methods (in green). However, the accuracy of MEEG localization (in red) using cMEM was quite robust to the number of EEG electrodes involved, reaching excellent performances (median AUC >0.8) even when only 20 EEG electrodes were added to the 272 MEG sensors. Figure 8b presents the distribution of SD values obtained on 100 static simulations, when decreasing the number of EEG electrodes. cMEM on MEEG showed the smallest SD values suggesting a more accurate sensitivity to the spatial extent, whatever was the number of EEG electrodes considered. These results are suggesting that the addition of only 20 EEG electrodes to the 272 MEG sensors will be sufficient to bring relevant information in the fusion, thus providing localization with good spatial accuracy.

**Fig. 9:**
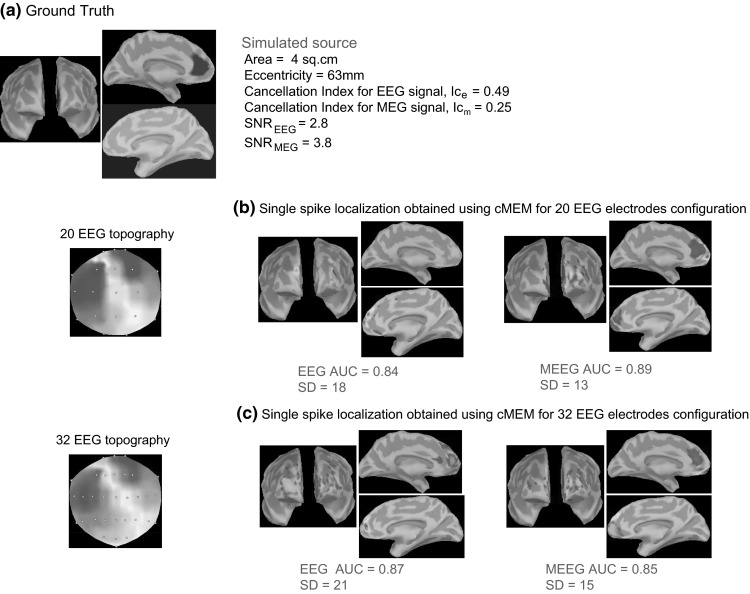
Qualitative assessment to evaluate the impact of the number of EEG electrodes using static simulation presented in Fig. 7. Visual analysis of source localization results together with AUC and SD values for a single static simulated source with area = 4 cm^2^ and eccentricity 63 mm. **a** Theoretical simulated source. **b** Source localization results obtained using cMEM method for 20 EEG electrode configuration on EEG and MEEG data. **c** Source localization results obtained using cMEM method for 32 EEG electrode configuration on EEG and MEEG data

Figure 9 illustrates cMEM localization for the left deep cingulate source presented in Fig. 7, when considering two subsampled EEG electrodes configurations. Localization of this deep source was difficult as none of the configurations were able to recover accurately the deeper aspects of the source. The SD values showed that MEEG improved the localization, especially since any fusion configuration lead to lower SD values than EEG for the three EEG electrodes configurations (see Figs. 7b, 9). For EEG source localization, the maximum amplitude source was localized on the wrong hemisphere for all three EEG configurations. However, from Fig. 7b for the 54 EEG electrodes configuration, EEG localization improved as it was indeed able to find a strong source within the simulated patch along with the strong source on the opposite hemisphere. MEEG localization for the three EEG configurations involved more accurately the deeper aspects of this anterior cingulate source, with sources well localized on the left hemisphere, but with larger amplitudes towards the more superficial and fronto-polar vicinity of the generator. Note that some spurious sources in the left frontal neocortex were also localized.

### Performance of Fusion on Spatio-temporal Simulations

**Fig. 10:**
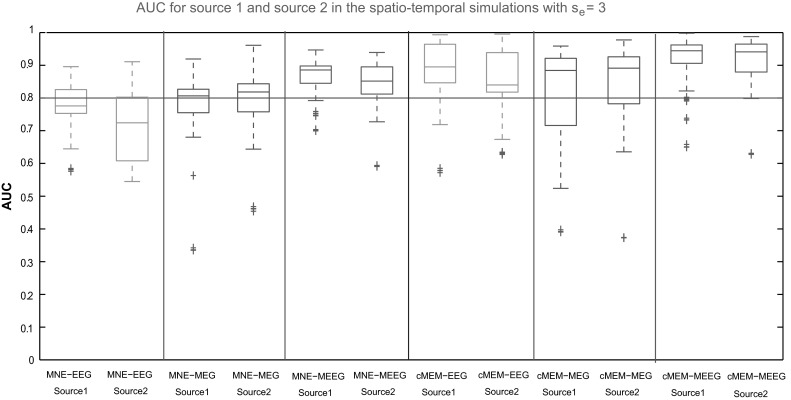
Evaluation of the source localization methods on the three modalities using AUC values over 100 spatio-temporal simulation configurations involving two randomly placed sources showing propagation within 15 ms duration between source 1 and source 2. *Boxplot* representation of AUC values for source 1 and source 2 with spatial extent *s*_*e*_ = 3. Color code for each modalities: EEG in *green*, MEG in *blue* and MEEG in *red* for the methods MNE and cMEM (Color figure online)

Figure 10 reports the distribution of AUC values obtained for source 1 and source 2 (at their respective peak, separated by a 15 ms delay) when using spatio-temporal simulations of propagating epileptic spikes. For each source, AUC distributions over 100 configurations are presented for cMEM and MNE methods and each modality. We observed that for all the modalities cMEM performed better than MNE in detecting the spatial extent of the propagating sources (higher AUC median values for both the sources when using cMEM). MEEG localization using cMEM provided the highest AUC values for both source 1 and source 2. EEG source localization was found slightly less accurate for source 2 than for source 1 (lower AUC median value). For both MEG and MEEG, similar level of detection accuracy was found for both sources. This could be explained by the fact that the electrical potentials of the two sources will further mix because of larger overlap of the topographies of the two sources in EEG for the given sensor arrays, which is less the case with the magnetic fields measured in MEG. Consequently, MEG and the information from MEG provided in the fusion helped to separate the two sources.

**Fig. 11:**
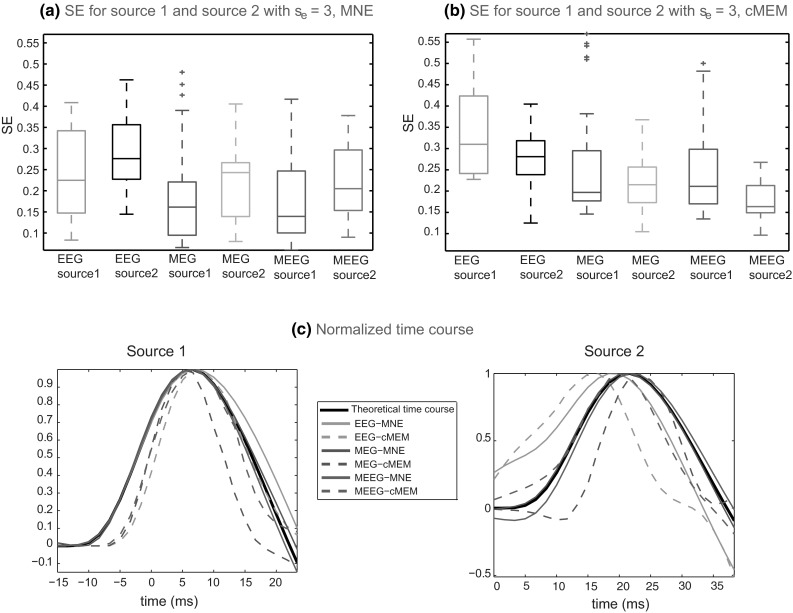
Evaluation of the source localization methods on the three modalities using SE estimates over 100 spatio-temporal simulation configurations involving two randomly placed sources showing propagation within 15 ms duration between source 1 and source 2. **a** *Boxplot* representation of SE values obtained for reconstruction of source 1 and source 2 using MNE method. **b** *Boxplot* representation of SE values obtained for reconstruction of source 1 and source 2 using cMEM method. Color code for each modalities: EEG in *green* for source 1 and *black* for source 2, MEG in *blue* for source 1 and *cyan* for source 2 and MEEG in *red* for source 1 and *magenta* for source 2. **c** Normalized mean time course of source reconstruction obtained for source 1 (*left* *plot*) and source 2 (*right* *plot*) using MNE and cMEM on EEG, MEG and MEEG data. Color code: *black* (*solid line*) for theoretical time course, EEG in *green*, MEG in *blue*, MEEG in *red*, *solid line* for MNE and *dashed line* for cMEM (Color figure online)

Analysis of the reconstructed time courses is shown in Fig. 11. We observed that SE was clearly smaller for MNE (Fig. 11a) than for cMEM (Fig. 11b) for both sources in EEG localization. For MEG and MEEG localizations, SE for MNE was still slightly smaller than SE for cMEM, but we found a clear improvement on cMEM SE for MEG and MEEG when compared to EEG. Moreover, MNE was able to reproduce the shape of the time course of first source better than the second source (larger SE for source 2). This could be explained by the fact that the SNR for source 1 was higher than source 2 since there was no mixing between the first and second source at the time of localization of source 1. The excellent performance of MNE in reconstructing the shape of the time course was rather expected, because MNE is a linear estimator. On the other hand, we provided here the first evaluation of the temporal behavior of cMEM localization. As cMEM sources consisted in non-linear estimates for each time sample independently, it was not obvious that it would reconstruct temporally smooth time courses. These first results are quite encouraging, especially for MEEG estimates providing almost similar temporal accuracy as MNE.

**Fig. 12:**
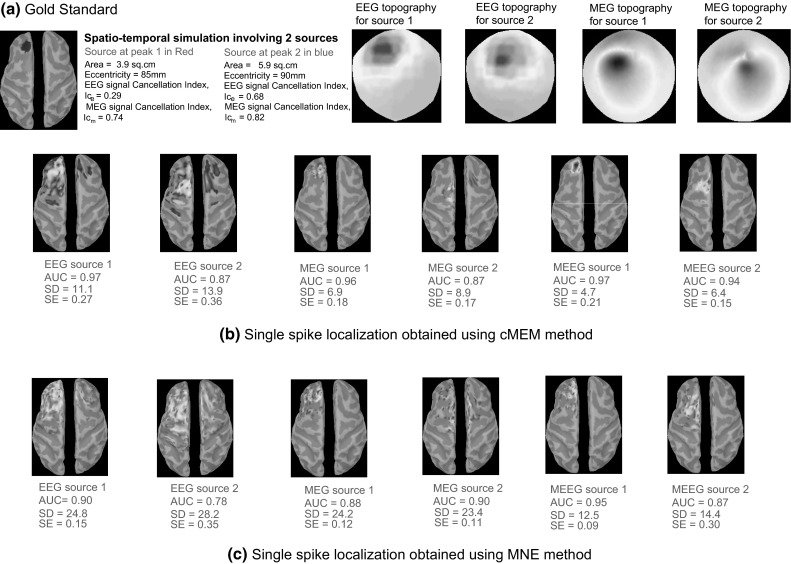
Qualitative assessment for example of spatio-temporal simulation. Visual analysis of source localization results together with AUC, SD, and SE values for an example of spatio-temporal simulation configuration. Source 1 with area = 3.9 cm^2^ and eccentricity 85 mm and Source 2 with area = 5.9 cm^2^ and eccentricity 90 mm. All source localization results are presented as the absolute value of the current density at the peak of the spike, normalized to its maximum activity and thresholded upon the level of background activity. **a** Theoretical simulated sources: area and eccentricity of the cortical source 1 and 2; associated EEG and MEG topography; Cancellation index for source 1 in EEG, *Ic*_*e*_ = 0.29 and in MEG, *Ic*_*m*_ = 0.74; Cancellation index for source 2 in EEG, *Ic*_*e*_ = 0.68 and in MEG, *Ic*_*m*_ = 0.82. **b** Source localization results obtained using cMEM on EEG, MEG and MEEG data. **c** Source localization results obtained using MNE on EEG, MEG and MEEG data

Figure 12 presents our results for a simulated spatio-temporal propagation from a left pre-frontal region to a left posterior superior frontal region. MNE and cMEM were able to localize accurately these two superficial sources, but with different sensitivity when recovering the spatial extents and the time courses. EEG localizations for both methods over-estimated the spatial extent by presenting large spatial spread around the true extent of the source (higher SD values than for MEG and MEEG). MEG localizations slightly under-estimated the spatial extent of the sources and also showed few distant spurious sources. This is probably due to the fact that the cancellation effect in MEG was very high (*Ic*_*m*_ = 0.78 for source 1 and 0.82 for source 2) and MEG was not able to recover the radial aspects of these generators. On the other hand, MEEG localizations provided a better estimation of the source spatial extent. From the visual inspection which is also in agreement with the metrics (Source 1: AUC = 0.97, SD = 4.7, and SE = 0.21; Source 2: AUC = 0.94, SD = 6.4, and SE = 0.15), MEEG localization using cMEM provided the most accurate detection of the sources with their respective spatial extents and time courses. The normalized mean time courses of source reconstruction for these two sources are presented in Fig. 11c. We observed that MNE was the most accurate in reconstructing the time course of source 1 (in green, blue and red solid lines for EEG, MEG and MEEG respectively). This behavior is in agreement with the lowest SE values (SE <0.15) estimated for source 1 when using MNE (Fig. 12). SE for source 2 using MNE and cMEM were the highest (SE >0.35) in EEG localization, which is also evident from the shape of the reconstructed time course in Fig. 11c. Both MNE and cMEM were able to recover the time courses of the two sources better in MEEG than EEG or MEG (Fig. 11c). Note that for MEEG, cMEM provided very accurate time course reconstructions around the peaks of source 1 and 2, whereas the amplitude decreased faster than MNE for lower SNR signals more distant from the peaks, illustrating the ability of cMEM to shut down the parcel.

#### Robustness to Model-Error

**Fig. 13:**
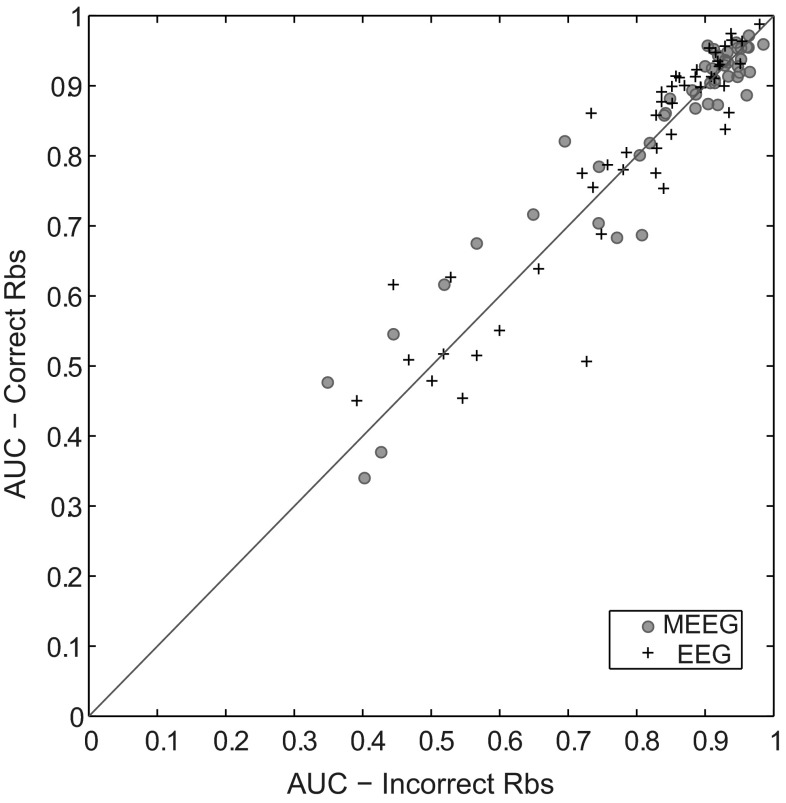
Test for robustness to model-error in simulation protocol. Plot showing the effect on localization accuracy when using correct Brain-to-skull conductivity (Rbs) ratio versus incorrect Rbs on EEG and MEEG data using cMEM method: EEG (*black plus sign*) and MEEG (*green circle*) (x-axis: AUC value for incorrect Rbs, y-axis: AUC value for correct Rbs) (Color figure online)

Figure 13 presents the effect on localization accuracy when using correct Rbs versus incorrect Rbs on EEG (black plus signs) and MEEG (green circle) data using cMEM method. We found that the cMEM method is robust to this mis-modelling in the simulation protocol as the localization accuracy when using incorrect Rbs in the EEG head model does not differ much from results obtained when using correct Rbs. In a recent study, (Wang and Ren 2013) tested the effect of correct and incorrect Rbs using simulations of EEG data when adding background noise or not. They showed that despite using the same Rbs in the EEG head model for simulation and localization there still exist localization errors in EEG source localization. This error was caused by contamination of the EEG data with background noise. This supports our simulation protocol where we added real background noise to both EEG and MEEG data.

### Application of cMEM Fusion Approach on Clinical Data

**Fig. 14:**
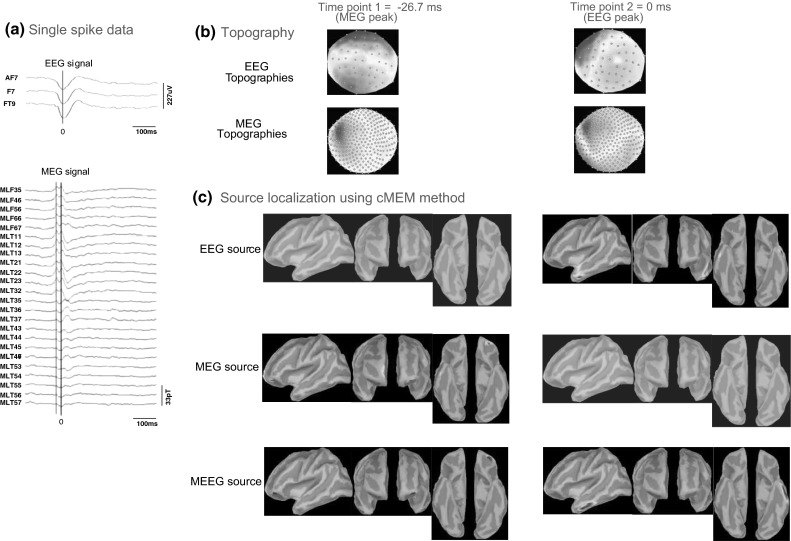
Patient 1—single spike localization. **a** EEG and MEG signal for the respective spike type (*vertical black line* = 0 ms in time, *red* *line* is the respective time point for selected EEG or MEG peaks). **b** EEG and MEG topographies for time point T1 (MEG peak) and T2 (EEG peak). **c** Source localization results using cMEM method for EEG data, MEG data and MEEG data

For patient 1, we identified six left fronto-temporal spikes fulfilling our selection criteria. Source localization was performed on each of these single spikes and results from all the spikes were then averaged (Supplementary Figure S1). Fig. 14 presents one of the single spike source localization results on EEG, MEG and MEEG data obtained using cMEM. For each spike, we identified two peaks in MEG (the first MEG peak occurring 26.7 ms before the second MEG peak) and one in EEG (second MEG peak was synchronous with the EEG peak). All single spike source localizations demonstrated propagation of activity from the left orbitofrontal region (at time point 1 = −26.7 ms, MEG peak) to the left temporal neocortex (time point 2 = 0 ms, EEG/MEG peak) in MEEG localizations. In MEG localizations, we observed the left orbitofrontal source along with a right fronto-mesial source at time point 1. On the other hand, EEG localizations (at time point 2, EEG peak) found mainly a left temporo-polar source while presenting also a right temporal source. When averaging the localization of the six spikes (Supplementary Figure S1), we found mainly the left orbito-frontal source in MEG at time peak 1, a left temporal neocortical source in EEG at time peak 2, while MEEG fusion described nicely the propagation between these two regions, suggesting the benefit of integrating EEG and MEG data using cMEM. The clinical seizure semiology of this patient suggested that the seizures originated from the left frontal lobe. Left fronto-temporal IEDs were recorded in EEG and MEG. This propagation from orbito-frontal to temporal neocortex identified by MEEG using cMEM is quite a plausible pattern of propagation for this type of epilepsy, following a well-known white-matter connection pathway.

**Fig. 15:**
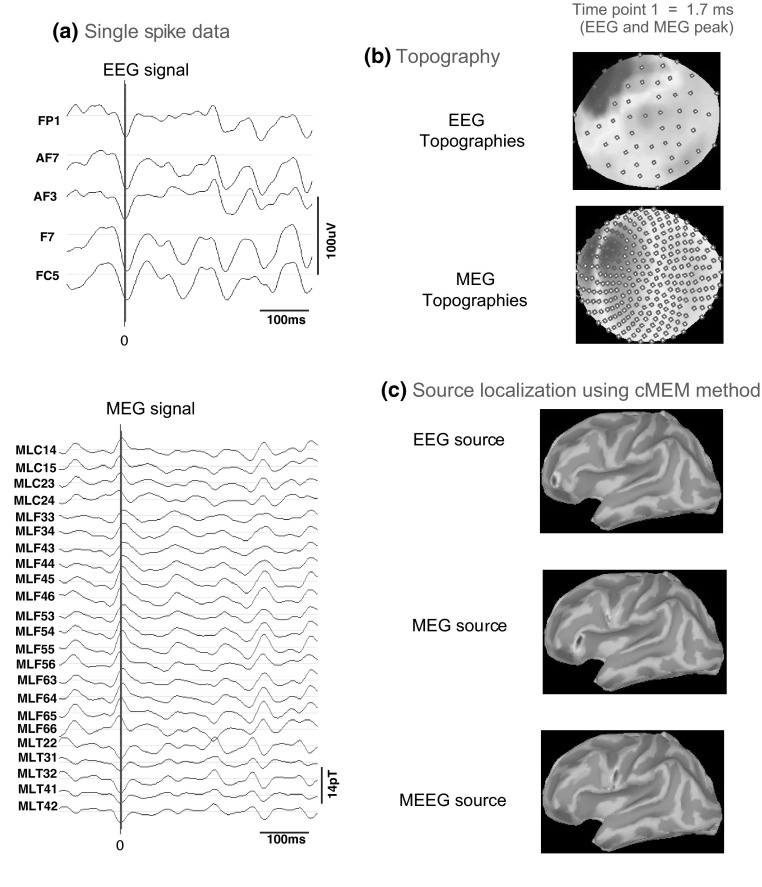
Patient 2—single spike localization. **a** EEG and MEG signal for the respective spike type (*vertical black line* = 0 ms in time, *red* *line* is the respective time point for selected EEG or MEG peaks). **b** EEG and MEG topographies for time point 1 (EEG peak and MEG peak). **c** Source localization results using cMEM method for EEG data, MEG data and MEEG data (Color figure online)

For Patient 2, we identified four left frontal spikes fulfilling our selection criteria. Single spike localizations were performed on these four spikes and then average of these four source localization results were obtained. In all the four single spike localization results (Fig. 15), we noticed that EEG localization found a left frontopolar source, whereas, MEG localization presented mainly two sources: one in the left inferior frontal gyrus and another in the inferior part of the left pre-central gyrus. However, MEEG fusion identified the main source in the inferior part of the left pre-central gyrus but with a slightly different spatial distribution than MEG pre-central source. The average of four single spikes localization (Supplementary Figure S2) reproduced similar results as seen in each single spike, suggesting good reproducibility. These results are rather interesting, since MEEG identified mainly a source in the inferior part of left pre-central gyrus, that was in perfect overlap with the FCD of the patient, whereas sources identified by EEG or MEG did not overlap with the anatomical lesion. The clinical seizure semiology of this patient also suggested an involvement of the inferior central region.

## Discussion

The purpose of this study was to propose and validate a new symmetrical EEG/MEG fusion strategy using the MEM framework. We provided an extensive evaluation of MEEG fusion when localizing single, non-averaged, epileptic spikes, using either realistic simulations or clinical data. Our results demonstrated the robustness of MEM-based fusion approaches to low SNR conditions of single spike localization and when recovering spatio-temporal propagations of epileptic discharges.

### Why Applying Fusion to Single Spike Localization?

For EEG and MEG to detect IEDs from background activity, the underlying generators should be spatially extended (Mikuni et al. 1997; Tao et al. 2007; Huiskamp et al. 2010). Although, single dipole fitting is currently the most common and clinically accepted method for the purpose of epileptic focus localization (Bast et al. 2004), distributed source models are more suitable for localizing the spatially extended generators of IED (Tanaka and Stufflebeam 2014). When localizing IEDs, several epileptic spikes showing a similar morphology and field maps are usually averaged to improve the SNR and then source analysis is performed on the averaged spikes (Bast et al. 2004; Hara et al. 2007; Tanaka et al. 2010). Several studies (Bast et al. 2004, 2006) explored the pros and cons of averaging spikes and suggested that averaging will confound any important spatio-temporal information present in each individual spikes due to cancellation of signals. Therefore, spatio-temporal source analysis of single spike will be more appropriate to provide information on the spike onset and propagation pattern by creating a balance between increasing SNR and spike variability (Tanaka et al. 2014). Moreover, single spike analysis of combined EEG and MEG recordings is favorable to take full benefit of the complementarities between these two modalities (Pataraia et al. 2005).

### Why cMEM Based Fusion Approach?

With the present study, we were able to show that single spike analysis using cMEM on EEG/MEG fusion data improved the spatial accuracy of spatially extended source reconstruction.

Symmetrical fusion of EEG and MEG within the MEM framework took place at three levels: (1) normalization and concatenation of the data and lead field matrices, (2) data driven parcellization, and (3) initialization of the probability of activation of each parcels. As a first step, the data and the lead field matrices of each modality were normalized by the standard deviation of the respective background activity, using the SNR transformation method described in (Fuchs et al. 1998) and (Ding and Yuan 2013). Different normalization methods have been proposed in previous works for combining EEG and MEG data. The motive behind using the SNR transformation method in our study was to account for the different physical units of MEG (Tesla) and EEG (Volt) and for their different noise content. Therefore, this modality-specific normalization seems appropriate for multimodal fusion of EEG and MEG. Most of other EEG/MEG fusion approaches differed in the way data were normalized and concatenated before applying the inverse operator. Some of the proposed methods consist in channel-wise SNR transformation (Fuchs et al. 1998), incorporation of intermodal noise covariance (Ko and Jun 2010), minimization of mutual information for channel selectivity (Baillet et al. 1999), row normalization of lead-field matrices, weighted normalization (Hong et al. 2013), and integration within a Bayesian framework (Henson et al. 2009). Note that we have tested our simulations with both global and channel-wise SNR transformation and there is no significant difference in the final result of fusion. However it is important to mention that a more accurate noise covariance model was taken into account during the MEM optimization process, rather than starting by a pre-whitening of the data as it is usually considered. In the present study, the noise covariance model was estimated as diagonal but with a different value for each channel, thus taking into account the noise level of each individual channel.

However, the second and third levels described in the present MEM fusion framework are specific to our proposed method. We believe that using fusion MSP scores (${\mathbf{MSP}}_{MEEG}$) for the whole cortex parcellization and for the initialization of the probability of each parcel to be active played an important role in combining the complementary information from EEG and MEG in the fusion process. In Eq. 12, we estimated ${\mathbf{MSP}}_{MEEG}$ using a logical OR operator to integrate ${\mathbf{MSP}}_{EEG}$ and ${\mathbf{MSP}}_{MEG}$ maps. Note that other fusion strategies could have been investigated at this level as well, as for instance using minimized mutual information for each source (proposed in (Baillet et al. 1999)) to reduce the redundancy between the two modalities.

### Static Simulations of Realistic IEDs

Using AUC metric to assess the detection accuracy of the source localization methods, we have demonstrated an overall higher spatial accuracy of MEEG localization when compared to the mono-modal localizations for all the evaluated methods (cMEM, MNE, dSPM and sLORETA). We also observed that the single spike localization of MEEG data improved the detection accuracy of the sources at all eccentricities when compared to EEG or MEG localizations (Fig. 5). This suggests that deeper sources can be localized more accurately with the fusion due to the increase in the number of recording channels and fusion of complementary information from EEG and MEG. We indeed showed that EEG data were likely to be more sensitive to deeper sources than MEG data measured using gradiometers, whereas MEEG fusion provided most accurate results.

SD seems an interesting metric for the evaluation of EEG, MEG and MEEG localizations. SD is influenced by both the spatial spread around the source and the presence of spurious sources. In Figs. 4a, b and 8b, we noticed that all the methods provided overall lower SD values for MEEG localization when compared to MEG and EEG localizations while cMEM performed better than MNE, dSPM and sLORETA for all modalities. This indicates that MEEG localizations presented less spatial spread of the solution around the true extent of the source or less spurious activities distant from the true source than EEG or MEG localizations. The simulation model used in this study involves a static patch of uniform activity, which has been extended to simulate different spatial extents of the source. In this model, the patch extends in all direction with uniform intensity, which is not fully realistic. This can indeed be a drawback, especially for MEG, when the patch included two opposing walls of sulcus leading to an increased amount of signal cancellation and low SNR signal. EEG simulated signals showed overall higher SNR due to the contribution of gyral sources. Therefore, most of the sources simulated in this study provided lower SNR for MEG simulated signals than for EEG simulated signals. This simulation bias explains the large variance observed in the distribution of SD values in MEG localizations; especially showing long tails towards large SD values (see one example in Fig. 6). We also checked that most results involving large SD values corresponded to simulations exhibiting a low SNR (deep sources or large cancellation effect).

### Impact of the Number of EEG Electrodes for Fusion

Scalp EEG is sensitive to both radial and tangential components of the sources, whereas MEG is mainly sensitive to the tangential components of the sources (Hämäläinen et al. 1993). As a result, in addition to the spikes seen by both modalities, it is not rare to detect EEG spikes where no MEG spikes are visible and vice versa (Iwasaki et al. 2005; Knake et al. 2006; Ramantani et al. 2006; Kakisaka et al. 2013). Spike visible on EEG only are explained by the better sensitivity of EEG to deeper and radially oriented source. Spikes visible on MEG only are explained by the sensitivity of MEG to mainly tangentially oriented sources and less influence of the skull resistivity leading to better SNR of MEG signal for sources in superficial, neocortical areas (Goldenholz et al. 2009; Huiskamp et al. 2010; Kakisaka et al. 2013). It would therefore be important to consider fusion of both modalities even when the spike is detectable on only one of the two modalities (Zijlmans et al. 2002). With fusion, we could probably improve these conditions where the spike is at low SNR in one of the modality but this was out of the scope of this study and will be considered in further studies. Difference in the EEG and MEG source analysis results can also be explained by the difference in the number of measurement sites between EEG and MEG. Most MEG systems are equipped with more than 100 sensors uniformly distributed around the whole head, which provides high spatial sampling. On the other hand, when recording EEG data only, high density montages involving 64, 128 or 256 channels are needed to ensure reliable EEG source analysis (Lantz and Grave de Peralta 2003; Babiloni et al. 2009; Brodbeck et al. 2011; Yamazaki et al. 2013). However, most clinical centers commonly use the conventional 10–20 EEG system for recording epileptic patients, which lacks the high spatial sampling required for the improved localization accuracy in EEG (Zelmann et al. 2013).

Analysis of combined EEG and MEG measurements from simultaneous recording was suggested to bring additional information missed by either modalities (Stefan et al. 1990; Fuchs et al. 1998; Iwasaki et al. 2005; Sharon et al. 2007; Babiloni et al. 2009). But, recording simultaneous EEG and MEG data is time consuming to set-up many EEG electrodes and can be associated with some discomfort for the subject wearing the EEG cap inside the MEG helmet, thus limiting the duration of the acquisition. We were able to show that MEEG localization using cMEM was quite robust to the number of EEG electrodes involved, reaching excellent performances (median AUC >0.8 and median SD values <10) even when only 20 EEG electrodes were added to the 272 MEG sensors (Fig. 8). These results suggest that the addition of only 20 EEG electrodes to the 272 MEG sensors, making sure that these electrodes were covering the lower aspects of both temporal lobes, will be sufficient to bring relevant information for the fusion, thus providing localization with good spatial accuracy. However, the example in Figs. 7b and 9 showed that all the 54 EEG electrodes were needed for recovering the deeper aspects of the source even in fusion. This could be explained by the fact that MEG performs poorly in detecting deep source locations in medial areas such as cingulate gyrus (Molins et al. 2008). Therefore, for most sources only 20 EEG electrodes in the fusion were sufficient but for few other sources the addition of well-placed EEG electrodes might be needed to cover the sites of interest. This raises an important question whether what are the best positions of EEG electrodes such that EEG’s information about the deeper and radially oriented sources can be effectively added to the MEG information in fusion. This point will be addressed in further details in a subsequent study but was out of the scope of this one.

### Spatio-temporal Simulations of Realistic IEDs

Assessing neuronal propagation during interictal spikes may take benefit from spatio-temporal source analysis of EEG and MEG data (Hara et al. 2007; Tanaka et al. 2010, 2014). Using dSPM (Shiraishi et al. 2005; Hara et al. 2007) and MNE (Tanaka et al. 2014), previous studies investigated the spatio-temporal source reconstruction of propagated MEG spikes. Although they based their results on averaged spikes localization due to the difficulty in localizing the low SNR individual spikes, it is more reliable to perform single spike localization to recover accurate information on the spike onset and propagation (cf. “Why applying fusion to single spike localization?” section). In addition, by combining simultaneously occurring EEG and MEG spikes, the SNR for individual spikes can be increased and complementary information from both modalities will lead to better representation of the propagation patterns (Bast et al. 2004). Therefore, in the present study, simulations of two spatially extended propagating sources, with overlapping time courses, were used to assess the performance of MEEG localization using cMEM. We observed that MEEG localization using cMEM provided the highest detection accuracy for both source 1 and source 2 (Fig. 10). Because of the overlap of topographies of the two sources in EEG, detection accuracy of source 2 was lower than source 1 in EEG localizations for both MNE and cMEM. On the other hand, MEG localizations provided similar detection accuracy for both sources due to smaller overlap between the topographies of the two sources. MEEG localization using MNE behaved similarly to EEG localization in detecting source 2 indicating the influence of spatial blurring effect of EEG in the fusion. Interestingly, MEEG localization using cMEM showed good performance in separating the two sources with the help of additional key information brought by MEG that was nicely taken into account with the MEM fusion framework (Figs. 10, 12). This shows that the fusion of EEG and MEG within the MEM framework is able to improve upon the spatial resolution of EEG localization due to the complementarities of the two modalities.

In this study, through shape error metric (“Validation metrics” section), cMEM reconstructed time courses were evaluated for the first time. cMEM being a non-linear localization procedure applied independently and iteratively on each time sample of the data, the reconstruction of smooth time courses was not obvious, as opposed to MNE that consists in applying a linear projector to the data. While MNE provides excellent accuracy in reconstructing the shape of the time courses of spatio-temporal overlapping sources, it was an important finding that cMEM estimates for MEEG data were able to provide very good accuracy as well (Fig. 11).

The main interest of this study was the fusion of EEG and MEG data within the MEM framework and comparison of cMEM method with MNE as the reference method was sufficient for this study. To address the issue of bias towards superficial sources known in MNE, we also included in our evaluation two noise-normalized variants of MNE: dSPM and sLORETA. Based on the results on static simulations, we concluded that despite the depth weighting property of dSPM and sLORETA, cMEM still provided an overall better spatial accuracy than dSPM and sLORETA, especially in the context of recovering source spatial extent. We did not provide a comparison of the cMEM method with the previously compared Hierarchical Bayesian methods (namely, Independent and Identically Distributed model-IID and spatially Coherent model—COH) as proposing MEEG fusion in this Bayesian framework was not the purpose of the study. However, in a recent paper from our group (Heers et al. 2015), we demonstrated the excellent performance of cMEM when compared to IID and COH, evaluating EEG/MEG source localization of IEDs on 15 patients, using intracranial EEG as a reference. Whereas we are fully aware that analysis using realistic simulations suffers from some bias, these recent results demonstrated the applicability of our methods on real data. Moreover, following a similar strategy than the one proposed by (Wang and Ren 2013), we showed that EEG and MEEG source localization using cMEM method was robust to the model-error introduced in the simulation protocol, and especially errors in brain-to-skull conductivity ratios. Currently, studies are in progress (Chowdhury et al. 2014) based on improved simulation paradigms: realistic simulations generated by neural mass model (Cosandier-Rimélé et al. 2010) and comparison of cMEM with other non-linear method such as 4-ExSo-MUSIC (Birot et al. 2011). Different variants of the MEM approach are now available for users as a toolbox (namely, BEst: Brain Entropy in space and time) in the Brainstorm software (Tadel et al. 2011), and the tutorial introducing this toolbox can be found here.^3^

### Performance of Fusion on Clinical Data

A detailed clinical validation of cMEM fusion was out of our scope and will be considered for future studies. However, we illustrated the behavior of cMEM fusion on two clinical cases. For patient 1, MEEG localization found mainly the propagation of activity from left orbito-frontal to left temporal neocortex when MEG found mainly the orbito frontal and EEG found the temporal neocortex activity. This is interesting to see that we were able to find clear propagation pathway between the frontal lobe and the ipsilateral temporal lobe only when using MEEG localizations. Such reproducible findings on few single spikes suggest a good accuracy of the fusion cMEM method. However, for the purpose of providing clinically useful results, the consensus between many spikes should be certainly investigated. Recently, (Aydin et al. 2015) showed that combined EEG-MEG source analysis reveals the propagation pathways in complete agreement to stereo—EEG (sEEG), while single modality EEG or MEG might only be sensitive to complementary parts of the epileptic activity. A study using Diffusion Tensor Imaging (Lin et al. 2008) described the connection between the anterior temporal lobe and the inferior frontal lobe to be mediated by the uncinate fasciculus (Makris and Pandya 2009); thus supporting a well-known anatomical substrate for the propagation patterns identified for patient 1. Generally ipsilateral cortical propagation occurred within 30 ms (Zumsteg et al. 2006); which was also what we noticed in the propagation pattern presented in patient 1 (within 26.7 ms). It was shown in (Tanaka et al. 2010) that spatio-temporal analysis of averaged MEG data provides more accurate information on spike propagation than averaged EEG data. This was consistent with our findings in patient 1, even though we did not localize averaged data but we presented the average of six single spike localization results. The propagation pattern was not found by EEG localization but both the primary (orbitofrontal) and secondary (temporal neocortex) source were found in the average of MEG localization results (Supplementary Figure S1). It was shown in (de Jongh et al. 2005) that the SNR of MEG is higher than EEG for frontal areas so MEG yields more spikes than EEG for frontal lobe epilepsy. The lower SNR spikes in EEG for frontal areas may explain why it was difficult to localize the orbito-frontal onset when using EEG only.

For Patient 2, MEEG using cMEM identified mainly a source in the inferior part of the left pre-central gyrus, which was in perfect overlap with the FCD of the patient. On the other hand, EEG and MEG localization identified mainly frontal sources which were probably secondary sources. A source closely related to the FCD was identified with MEG only on single spike localization. However, only MEEG enhanced the generators in the lesion as the primary source with largest amplitude. (Bast et al. 2004) investigated nine patients with localization-related epilepsy and FCD, and showed that it was important to average the EEG and MEG spikes from lesional zone to obtain an accurate localization of the MRI-defined lesion (Bast et al. 2004). (Heers et al. 2012) showed that the localization of averaged interictal MEG spikes was useful in locating subtle MR imaging abnormalities showing peri-insular lesion. (Hisashi Itabashi 2014) studied six patients with FCD and showed that source localization of averaged EEG and MEG spikes can confirm the existence of abnormalities associated with FCD based on MR imaging. On the other hand, we showed that localization of single spike of MEEG data found the origin of the spike consistently within the FCD lesion in patient 2. This confirms the advantage of localization of combined EEG and MEG data even in low SNR conditions. This is also in complete agreement with a recent study (Aydin et al. 2015), which investigated the contribution of combined EEG/MEG in comparison to single modality EEG or MEG source analysis of the epileptic activity using a dipole scanning approach. They validated their results with sEEG, where no major dipole cluster was noticeable neither with EEG nor with MEG around the active contacts in sEEG, while there were clear clusters around the active contacts in MEEG. They showed that MEEG localizations were not simply the union of EEG and MEG results but a rather complex interplay of both modalities compensating their relative shortcomings.

## Conclusion

In this paper, we proposed symmetrical fusion of EEG and MEG within MEM framework as a novel method for localizing the onset and propagation patterns of spatially extended generators of IEDs. Effective integration of the complementary information from EEG and MEG in cMEM was demonstrated based on realistic simulations and illustrated on real epileptic data. Overall, for both mono-modal and multimodal data we noticed better performance of cMEM than MNE, dSPM and sLORETA in detecting the spatially extended and propagating sources. Our findings suggest that it is better to perform EEG-MEG fusion when localizing single spikes using cMEM: (1) To yield better recovery of the source spatial extent. (2) To improve the sensitivity to source depth. (3) To represent better the spatio-temporal propagation patterns of the underlying generators of epileptic discharges. We also showed that the addition of only few EEG electrodes brings additional information missed by MEG, in order to allow an optimal EEG-MEG fusion.

### Electronic supplementary material

Supplementary material 1 Figure S1. Patient 1 - Average of six single spike localizations. (a) EEG and MEG signal for the respective spike type (vertical black line = 0 ms in time, red line is the respective time point for selected EEG or MEG peaks)). (b) EEG and MEG topographies for time point 1 (MEG peak) and time point 2 (EEG peak). (c) Source localization results using cMEM method for EEG data, MEG data and MEEG data (EPS 35727 kb)

Supplementary material 2 Figure S2. Patient 2 - Average of four single spike localizations. (a) EEG and MEG signal for the respective spike type (vertical black line = 0 ms in time, red line is the respective time point for selected EEG or MEG peaks)). (b) EEG and MEG topographies for time point T1 (EEG and MEG peak). (c) Source localization results using cMEM method for EEG data, MEG data and MEEG data. (EPS 14707 kb)

## Appendix: AUC Estimation

To assess how a source localization method could be sensitive to the spatial extent of the underlying generator, AUC metric was adapted by (Grova et al. 2006) to fit the context of a distributed source model, in order to take into account that there are quite more inactive dipolar sources than active sources in our simulation schemes.

This detection accuracy index is estimated when the Ground truth is available, where ROC curves are generated by plotting the sensitivity against the false positive detection rate for different detection thresholds, $(\beta \in [0,1])$. Normalized energy for both the estimated and the simulated current distribution were used to quantify the amount of true positive (TP), true negative (TN), false negative (FN), and false positive (FP) for each threshold $\beta$.

$$sensitivity(\beta ) = {{TP(\beta )} \mathord{\left/ {\vphantom {{TP(\beta )} {(TP(\beta ) + FN(\beta )}}} \right. \kern-0pt} {(TP(\beta ) + FN(\beta )}})$$

$$specificity(\beta ) = {{TN(\beta )} \mathord{\left/ {\vphantom {{TN(\beta )} {(TN(\beta ) + FP(\beta ))}}} \right. \kern-0pt} {(TN(\beta ) + FP(\beta ))}}$$

However, to interpret the area under the ROC curve as a detection accuracy index, one should provide the same number of active and inactive sources to the ROC analysis. Considering the *p* dipolar sources on the cortical surface, only few dipoles were actually active (*p*_*a*_) compared to the large number of inactive dipoles (*p*–*p*_*a*_). Therefore, selection of same number of inactive sources as the active sources is required. This was done by randomly selecting inactive sources among the *p*–*p*_*a*_ sources located within the immediate spatial neighborhood of the simulated source (**AUC**_**close**_) or among the local maxima of the reconstructed activity located far from the simulated source **(AUC**_**far**_**)**. Final **AUC** index was then computed as a mean of the **AUC**_**close**_ and **AUC**_**far**_.

## Footnotes

1 http://www.brainvisa.info.

2 The center of head was defined with the fiducial points marked during EEG/MEG acquisition. It is the point which is equidistant to the left and right peri-auricular points, at the same height of the location of the nasion.

3 http://neuroimage.usc.edu/brainstorm/Tutorials/TutBEst.
